# A comparative study on the nutrient and organic acid profiles of selected pepper genotypes

**DOI:** 10.1038/s41598-026-37078-w

**Published:** 2026-01-28

**Authors:** Peng Zhou, Dan Xing, Dehui Tu, Shiqing Peng, Jianwen He

**Affiliations:** Research Institute of Pepper, Guizhou Academy of Agricultural Science, Guiyang, 550025 Guizhou China

**Keywords:** Pepper varieties, Nutrient compositions, Organic acids, Principal component analysis, Biochemistry, Plant sciences

## Abstract

To comprehend the nutritional components and organic acids content of peppers for the purpose of advancing pepper breeding, it was imperative to analyze and compare the levels of nutritional components (capsanthin, capsaicin, dihydrocapsaicin, reducing sugar, total amino acids, crude fat, crude fiber and protein) as well as organic acids content. In this study, 18 varieties of peppers (*Capsicum annuum* L.) were assessed for their nutrient compositions and organic acids. variance analysis, principal component analysis (PCA), and hierarchical cluster analysis(HCA) were employed for data analysis. The levels of nutrient compositions, including capsanthin, capsaicin, dihydrocapsaicin, reducing sugar, total amino acid, crude fat, crude fiber and protein, varied within the ranges of 11.63–71.75 ug g^− 1^ DW, 0.15–6.86 mg g^− 1^ DW, 0.05–4.74 mg g^− 1^ DW, 0.73–2.54%, 9.53–34.48%, 7.23–19.50%, 19.77–32.64% and 9.96–5.30%, respectively. Concerning 64 organic acids, 11 organic acids(3-D-hydroxybutyric acid, 3-hydroxyphenyl-hydracrylic acid, maslinic acid, 2-indolecarboxylic acid, 3,4-dihydroxyphenylacetic acid, carnosic acid, ethylmalonic acid, maleic acid, methylmalonic acid, oleanic acid and sebacic acid) were not detected. L-malic acid, cis-lactic acid and succinic acid were the main organic acids in 18 pepper varieties, ranking first, second and third respectively. Their concentrations accounted for 13.7% − 66.8%, 37.3% − 54.1%, and 3.1% − 18.3% of the total organic acid content respectively. PCA and HCA revealed that all pepper varieties formed three category, respectively. These nutritional profiles and organic acid compositions could be instrumental in pepper breeding programs aimed at developing novel cultivars, thereby expanding options for both producers and consumers.

**Supplementary Information:**

The online version contains supplementary material available at 10.1038/s41598-026-37078-w.

## Introduction

Pepper (*Capsicum annuum* L.), known for its unique favors and varieties, remains high rates of consumption and economic value. Currently, it is extensively cultivated in the world as a spice enhances the flavor of food and serves as a key ingredient in numerous global cuisines. Moreover, Pepper is utilized in various applications, including medicines, cosmetics, natural colorants, and decorations purposes^1,2^. Additionally, pepper fruits are abundant in vitamins, minerals, phenolic compounds, natural antioxidants, and other nutritional components that are vital for human health. However, distinct pepper varieties exhibit variations in their nutritional profiles and metabolites^3,4^.

Many factors, including fruit maturity, environmental conditions, and cultivation techniques, likely influence the biochemical composition of fruit^5–8^. However, the primary source of variation is attributed to the genetic background^9^. Guizhou ranks first the nation for both production and sales of chili peppers, renowned for their distinctive mellow and spicy taste. Additionally, the region’s favorable geographical and climatic conditions create an ideal environment for production^9^, resulting in products with differentiated chemical composition and sensory (dark color and acid favor) characteristics^3,10,11^, which appeal to consumers across China. As awareness of healthy food increases, consumer interest in foods rich in antioxidants and nutritional quality also rises. breeding for enhanced fruit quality has emerged as an significant breeding objective for most pepper breeding programs, given the new niche in the marke^12^. Characterization of the pepper agro-biodiversity that exists in the collections can assist breeding programs, giving more insight on the fruit quality. It is essential to quantify parameters such as nutrient compositions alongside traditional quality attributes, including protein, capsaicin and organic acids et al. Organic acids play a crucial role in sensory characteristics and health benefits of products^13,14^. Different organic acids contributes distinct flavors and affect the acidity of the products^15–17^. Up to now, most research has focused on the appearance quality, some conventional nutritional indicators^18^, and volatile substances of peppers^19^. However, there are few reports on the research of organic acids in peppers^18,20^. A thorough germplasm characterization can provide insights for selecting of promising breeding lines, ultimately enhancing the fruit quality and nutritional value of future pepper varieties.

The research aimed to qualitatively and quantitatively analyze various nutrient and organic acids components including capsanthin, capsaicin, dihydrocapsaicin, reducing sugar, total amino acid, fat, protein and 64 organic acids in 18 peppers for evaluating their correlated genetic variation through principal component, and hierarchical cluster analyses to assist breeding and the development of new pepper varieties for industrial and market applications.

## Materials and methods

### Sample collection and preparation

18 varieties of peppers (Table 1) were hand-harvested at optimum ripeness in autumn 2023 and used for this study. These peppers were cultivated under standard field conditions and supplied by the Research Institute of Pepper, Guizhou Academy of Agricultural Science. Following harvest, the samples were dried and crushed into powder and then stored in a the freezer at −40 ℃ before analysis.

**Table 1:** Information of 18 pepper varieties.

Number	Varieties	Regions(in China)
Y1	Dafang zhoujiao	Dafang, Guizhou
Y2	Zunyi chaotianjiao	Zunyi, Guizhou
Y3	Huaxi lajiao	Guiyang, Guizhou
Y4	Baiyi lajiao	Guiyang, Guizhou
Y5	Suiyang lajiao	Zunyi, Guizhou
Y6	Niuchang lajiao	Liupanshui, Guizhou
Y7	Meijiang mingzhu	Zunyi, Guizhou
Y8	Huang ping xianjiao	Kaili, Guizhou
Y9	Huangyang xiaomila	Zunyi, Guizhou
Y10	Linka lajiao	Guiyang, Guizhou
Y11	Pingtang lajiao	Qianan, Guizhou
Y12	Yutang xianjiao	Tongren, Guizhou
Y13	Yunganjiao	Kunming, Yunnan
Y14	Shizhuhong VI	Chongqin
Y15	Changjiao VIII	Shanxi
Y16	Sanyinjiao	Tuocheng, Henan
Y17	Rucheng xiaomila	Hunan
Y18	Xinjiao III	Xinjiang

### Nutritional compositions

#### Capsanthin

Capsanthin was extracted and detected according to the method of the reference reported^21^. 0.5 g of the sample was weighed and placed in a 10 mL centrifuge tube. Subsequently, 10 mL of acetone was added, and the mixture was dissolved ultrasonically at 35 °C for 30 min. After cooling to room temperature, the supernatant was extracted and filtered through a 0.22 μm nylon filter membrane. The filtrate was then injected into Agilent 1260 high-performance liquid chromatography(HPLC) system employing a ZORBAX Eclipse Plus C18-A column (250 × 4.6 mm, 5 μm) with a detection wavelength set at 474 nm and eluted using HPLC grade acetonitrile/water (V: V = 9:1) with rate of 1.0 mL min^− 1^, a column temperature of 30 °C, and an injection volume of 10 µL.

#### Capsaicin and Dihydrocapsaicin

Extraction and detection were carried out according to the procedure described by Li J et al.^18^ and the Chinese National Standard GB/T 40,348, 2021^22^. A 0.5 g sample was weighed with a precision of 0.001 g and transferred into a 10 mL centrifuge tube. Subsequently, 10 mL of a methanol and tetrahydrofuran mixture (V: V = 1:1) was added, and the mixture was subjected to sonication at 35 °C for 30 min. After cooling to room temperature, the supernatant was collected using a 0.22 μm nylon filter. The filtered supernatant was then injected into a liquid chromatograph (Agilent 1260 II) to determine the content of capsaicin and dihydrocapsaicin.

Capsaicin and dihydrocapsaicin were identified and quantified using a symmetric C18 column (ZORBAX Eclipse Plus C18-A 250 × 4.6 mm, 5-Micron), where 10 µL of the sample was injected at a flow rate of 0.4 mL min⁻¹, and the column temperature was maintained at 40 °C. The mobile phase consisted of solvents A (65% HPLC grade methanol) and B (35% pure water), with a detection wavelength of 280 nm. Compounds were identified based on the retention times of the standards and quantified according to the standard curve.

#### Reducing sugar

Reducing sugar analysis conducted following the method and the Chinese National Standard GB/T 5009.7, 2016^23^ and the reference^24^. 3.0 g sample of pepper powder was accurately weighed and transferred into a 100 mL beaker, where it was homogenized into a paste using a small amount of distilled water. Subsequently, 50 mL of distilled water was added to the beaker, and the mixture was thoroughly stirred before being subjected to leaching of reducing sugars in a constant temperature water bath at 50 °C for 20 min. Following this step, filtration techniques were employed to separate the solution from any solid residue. The residue was washed with an additional 20 mL of distilled water prior to another round of filtration. All supernatant obtained from both rounds was combined in a 100 mL volumetric flask and diluted with distilled water to the calibration mark. The combined supernatant was thoroughly mixed to ensure uniformity, serving as the liquid sample for subsequent measurement of reducing sugars.

Three numbered test tubes (25 mL) were prepared, and 2 mL of the sample containing reducing sugar was added to each tube. Subsequently, 1.5 mL of a reagent consisting of 3,5-dinitrosalicylic acid was introduced. Finally, the absorbance value for each individual tube was measured and reducing sugar was quantified according to the standard curve. The values were calculated as follows:

1 $$X(\% ) = \frac{{m \times {V_\tau }}}{{{m_o} \times {V_S} \times 1000}} \times 100$$

*X* represents tested sample, %; *m* is the amount of sugar(mg) obtained from the standard curve; *V*_*T*_ is extracted liquid volume (mL); *V*_*S*_ is the sample volume (mL) taken during the determination; *m*_*o*_ is the sample mass (g);

#### Total amino acid

Total amino acid content was carried out according to the procedure described by W XP et al.^25^. A 1.0 g sample and 5 mL of 10% acetic acid were added to a mortar. The mixture was then transferred to a 100 mL volumetric flask and diluted to 100 mL with pure water. The sample was filtered into a conical flask, and the filtrate was used for the determination.

A 4 mL sample of the liquid was measured and placed into a 25 mL volumetric flask. Pure water (4.0 mL), buffer (1 mL), and ninhydrin (1 mL) were added to the volumetric flask and shaken well. After heating in a boiling water bath for 15 min, the solution was cooled quickly to room temperature with cold water and then diluted to volume with pure water. The amino acid concentration was determined, and the values were calculated as follows:

2 $$X = \frac{{{C_S} \times 250}}{{W \times V}}$$

*C*_*S*_ is the amino acid concentration of the sample measured by the standard curve; *W* is sample weight (g); *V* is liquid volume to be measured (mL).

#### Fat and protein

The fat and protein content was determined according to the method described by OT Abiola^26^.

#### Crude fiber

The crude fiber was identified according to the procedure described by the method reported^29^. 0.5 g pulverized sample was weighed and placed into a constant-weight sealed filter bag. The sample underwent successive treatments including pickling, washing, alkali washing, and additional washing. The filter bag was then washed with ethanol and ether and dried to a constant weight.

For moisture content determination, 2.0 g samples were weighed in an aluminum box. The values were calculated as follows:

3 $$X(\% ) = \frac{{G \times 100}}{{m(1 - f)}}$$

*X* represents tested sample, %; *G* represents residual mass (or mass lost by high temperature furnace), g; *m* represents the mass of the sample, g; *f* represents the content of drying base moisture in the sample, %.

### Preparation of extracts and detection method for organic acids

The pepper sample was divided into three 50 mg subsamples. To each subsample, 0.5 mL ice-cold extraction solution (70% methanol and 0.1% formic acid) and 10 µg mL^− 1^ reserpine(internal standard for relative quantification) were added. The samples were vortexed and sonicated in water bath at room temperature for 15 min and then centrifuged at 12,000 r/min for 10 min. Finally, the supernatants were collected. The extraction process was repeated once. The supernatants were combined, filtered through a 0.2 μm nylon membrane and stored at −20 ℃ until analysis.

Organic acids were detected by Ultra Performance Liquid Chromatography-Mass Spectrometry(UPLC-MS) according to the method reported by Li, N et al.^30^. metabolit separation was achieved with an ACQUITY HSS T3 column (1.8 μm, 100 mm×2.1 mm i.d.). Mobile phases were ultrapure water with 0.05% formic acid (solvent A) and acetonitrile with formic acid(solvent B). The gradient program initiated at 95% A and 5% B(V/V) for 3 min, followed by 5% A and 95% B for 1.5 min, and then returned to initial elution conditions for 2.5 min at a flow rate of 0.2 mL min^− 1^. The injection volume was 2 µL and all samples were injected in duplicate.

Parameters for MS analysis were set using negative (−4500℃) and positive ionization mode (5500℃) with spectra acquired over a mass range of 50 to 1200 m/z. The parameters settings were as followed: capillary voltage at 3.5 kV and curtain gas at 35 psi. In the Q-Trap 6500, each ion pair was scanned according to optimized de-clustering voltage and collision energy.

### Data analysis

SPSS (IBM SPSS Statistics for Windows, v.22.0; IBM Corp., New York, NY, USA) was used for statistical analysis, and significant differences in nutrients composition and organic acids were studied via one-way analysis of variance (ANOVA) using Tukey’s test α = 0.05). Data normality was examined using Shapiro-Wilk tests (*n* < 50) and Q-Q plots. Homogeneity of variances was evaluated via Levene’s test. For normally distributed data with equal variances, one-way ANOVA followed by Tukey’s post-hoc test was used; otherwise, non-parametric equivalents (Kruskal-Wallis) were applied. The stacking histogram was drawn by Origin 2018 (Origin, Inc., San Francisco, CA, USA). In this experiment, nutrients compositions and organic acids of 18 pepper varieties were analyzed using principal component analysis (PCA) to clarify the relationships between the different pepper varieties assessed. Finally, heat map (with Pearson’s correlation coefficients as a distance measure) and hierarchical cluster analyses(HCA) were performed on all measured indicators to highlight and better explain the correlations between indicators and samples. PCA and HCA were performed using the Origin 21.

## Results

### Nutritional compositions

**Fig. 1:**
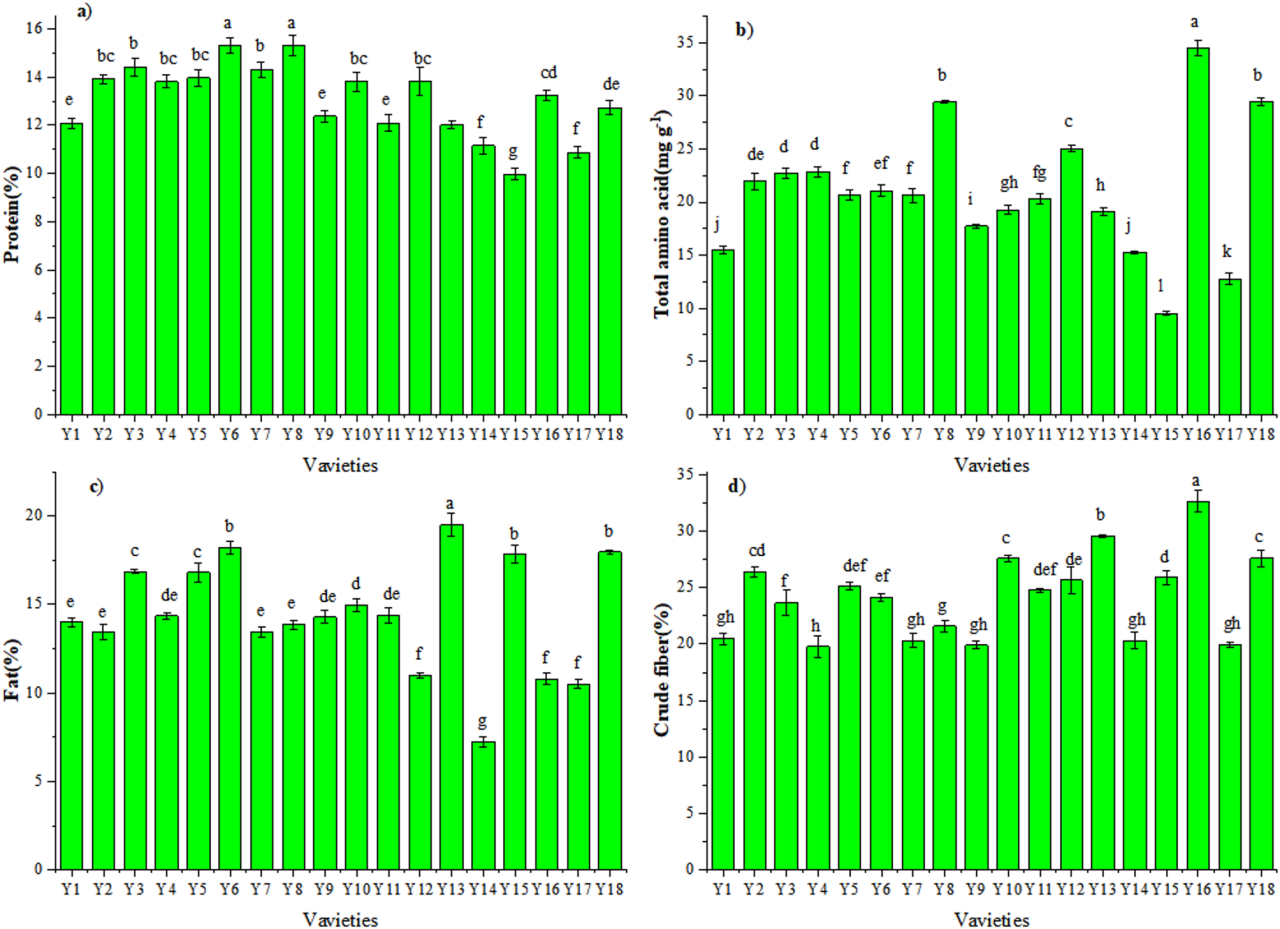
Contents of: **(a)** protein, **(b)** Total amino acid, **(c)** fat, and **(d)** crude fibre for the 18 pepper varieties. Y1-Y18 represent the numbers of 18 pepper varieties.

As shown in Fig. 1 and Table S1, among the 18 pepper varieties examined, Y6 and Y8 maintained a significantly higher protein (15.3%) than 16 other varieties. The protein content (Fig. 1a) in Y15 was the lowest (1.7%). No significant difference was found with (Y2, Y3, Y4, Y5, Y7, Y10, Y12), (Y16, Y18), (Y1, Y9, Y11, Y13, Y18). Total amino acid content (Fig. 1b) was significantly higher in Y16 (34.48 mg g^− 1^ DW) than in all other varieties. That of P3 (8.4 mg g^− 1^ DW) was significantly lower than all other varieties. There was no significant difference with (Y2, Y6), (Y3, Y4), (Y5, Y6, Y7, Y8, Y11), (Y10, Y11), (Y10, Y12), (Y1, Y14). The fat content (Fig. 1c) in Y13 (19.5%) was higher than all other varieties, and the lowest content was Y14 (7.23%). There was no significant difference with (Y6, Y15, Y18), (Y9, Y10, Y11), (Y1, Y2,Y4, Y11), (Y12, Y16, Y17). The crude fibre (Fig. 1d) reached its maximum in Y16 (32.64%), and relatively high values also observed in Y18(27.61%), Y13(25.58%), Y10(27.56%), whereas a minimum was recorded in Y7 (19.93%). No significant difference was found with (Y3, Y5, Y6, Y11, Y12, Y15), (Y2, Y10, Y18), (Y1, Y4, Y7, Y8, Y9 Y14, Y17).

**Fig. 2:**
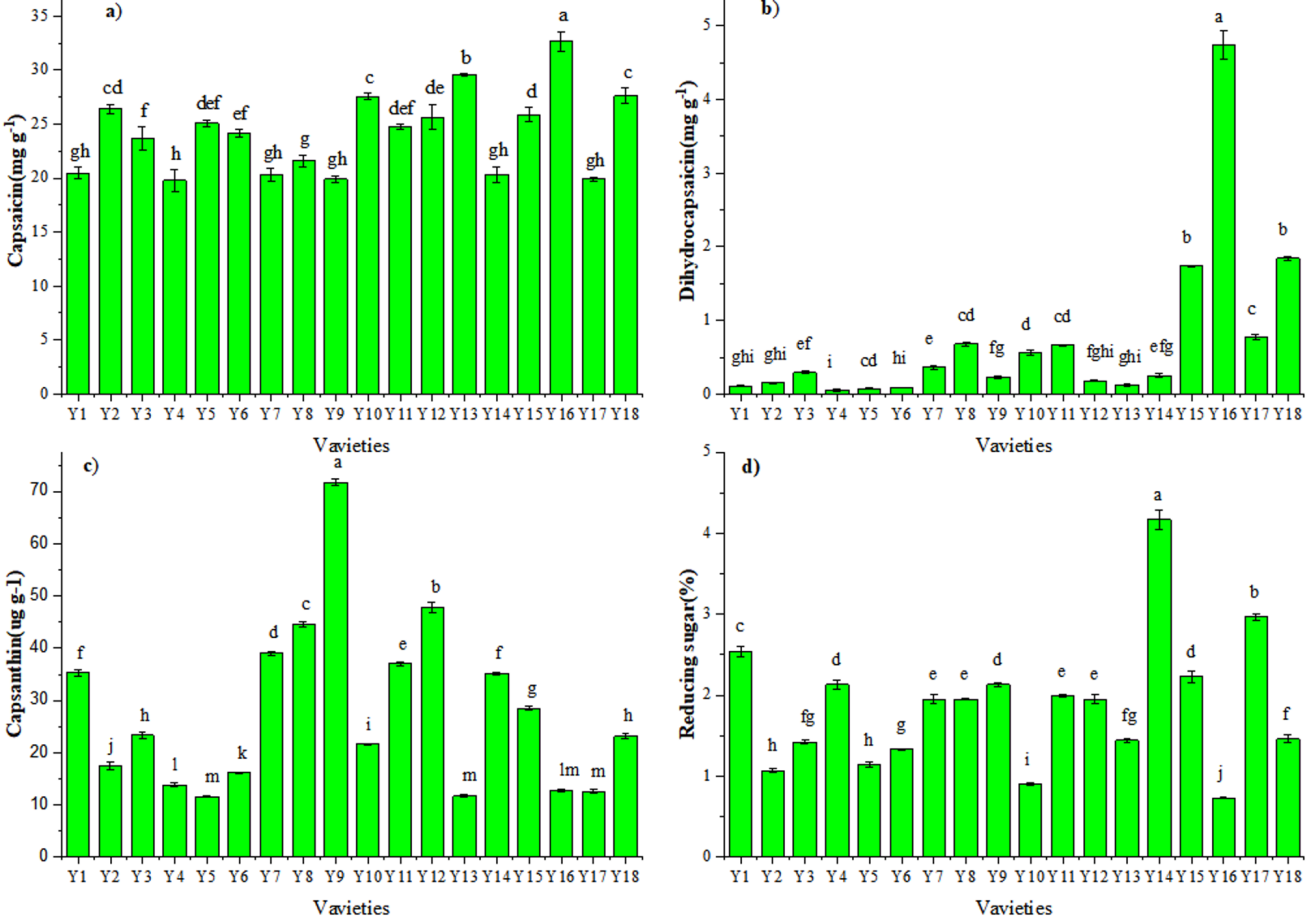
Content of: **(a)** Protein, **(b)** Total amino acid, **(c)** Fat, and **(d)** Crude fibre for the 18 pepper varieties.

Capsaicin and dihydrocapsaicin content varied significantly across the 18 pepper varieties (Fig. 2a and b), where content of Y16, Y15, Y18, and Y16 were comparatively greater. Capsaicin content peaked in Y16 (6.86 mg g^− 1^ DW and 4.74 mg g^− 1^ DW), followed by Y15 (4.10 mg g^− 1^), and there was a significant difference between them. Interestingly, dihydrocapsaicin content was also the highest in Y16 (4.74 mg g^− 1^ DW), followed by Y18 (1.84 mg g^− 1^ DW) which was not significant difference with Y15. Similarly, Capsaicin and dihydrocapsaicin content of other varieties were low except Y8, Y10 and Y11. Capsanthin ranged from 11.63 mg g ^− 1^ DW to 71.75 mg g ^− 1^ DW among the 18 varieties (Fig. 2c), with a maximum of Y9 (71.75mg g^− 1^ DW), followed by Y12 (47.73 mg g^− 1^ DW) and Y8 (44.56 mg g^− 1^ DW), while content of Y5, P13, P16 and P17 were low. There were significant differences in most varieties, except (Y1,Y15), (Y13,Y18), (Y5, Y13, Y16, Y17). Reducing sugar content was showed in Fig. 2d, Y14 (4.17%) maintained the highest content in the 18 pepper varieties, followed by Y17(2.97%), content of Y16 was lowest. A significant difference was observed between Y1, Y10, Y14, Y17 and other pepper varieties.

### Content of 64 organic acids

Several studies have proved that significant health risks and benefits are associated with dietary food choice^30^. Organic acids play a crucial in cancers, cardiovascular and cerebrovascular disorder^14^, while also contributing to the nutritional value and taste of food^31^.

The amounts and diversities of organic acids were found to differ significantly across peppers as a result of detection using LC–MS/MS, Among them, 11 types of organic acids were not detected in all the peppers (Table S2), including 3-hydroxybutyric acid, 3-hydroxyphenyl-hydroxypropionic acid, maslinic acid, 2-indole carboxylic acid, 3,4-dihydroxybenzoic acid, taurine, ethyl succinate, maleic acid, methyl succinate, oleic acid, and decanoic acid. Some organic acids only exist in some pepper varieties (Table S2). On average, L-malic acid, cis-aconitic acid, and succinic acid constituted the predominant organic acids, accounting for 13.7%−66.8%, 37.3%−54.1%, and 3.1%−18.3% of the all organic acids, respectively (Fig. S1-S18). The combined levels of L-malic acid, cis-aconitic acid, and succinic acid ranged from 77.5% to 86.7% across all samples.

Organic acid profiles varied markedly across different pepper cultivars (Table S2). Notably, 2-hydroxyisovaleric acid exhibited dramatic concentration disparity, with values spanning a wide range from 39.6 ng g⁻¹ DW to 489.44 ng g⁻¹ DW. Caffeic acid also showed substantial variability, its concentrations ranging from 80.29 to 462.48 ng g⁻¹ DW. Furthermore, several analytes, including salicylic acid, 4-hydroxyphenylacetic acid, and neochlorogenic acid, were undetectable in specific cultivars, which implied that the regulation of metabolic pathways governing organic acid synthesis may be genotype-dependent.

### PCA of nutritional compositions and organic acids

**Fig. 3:**
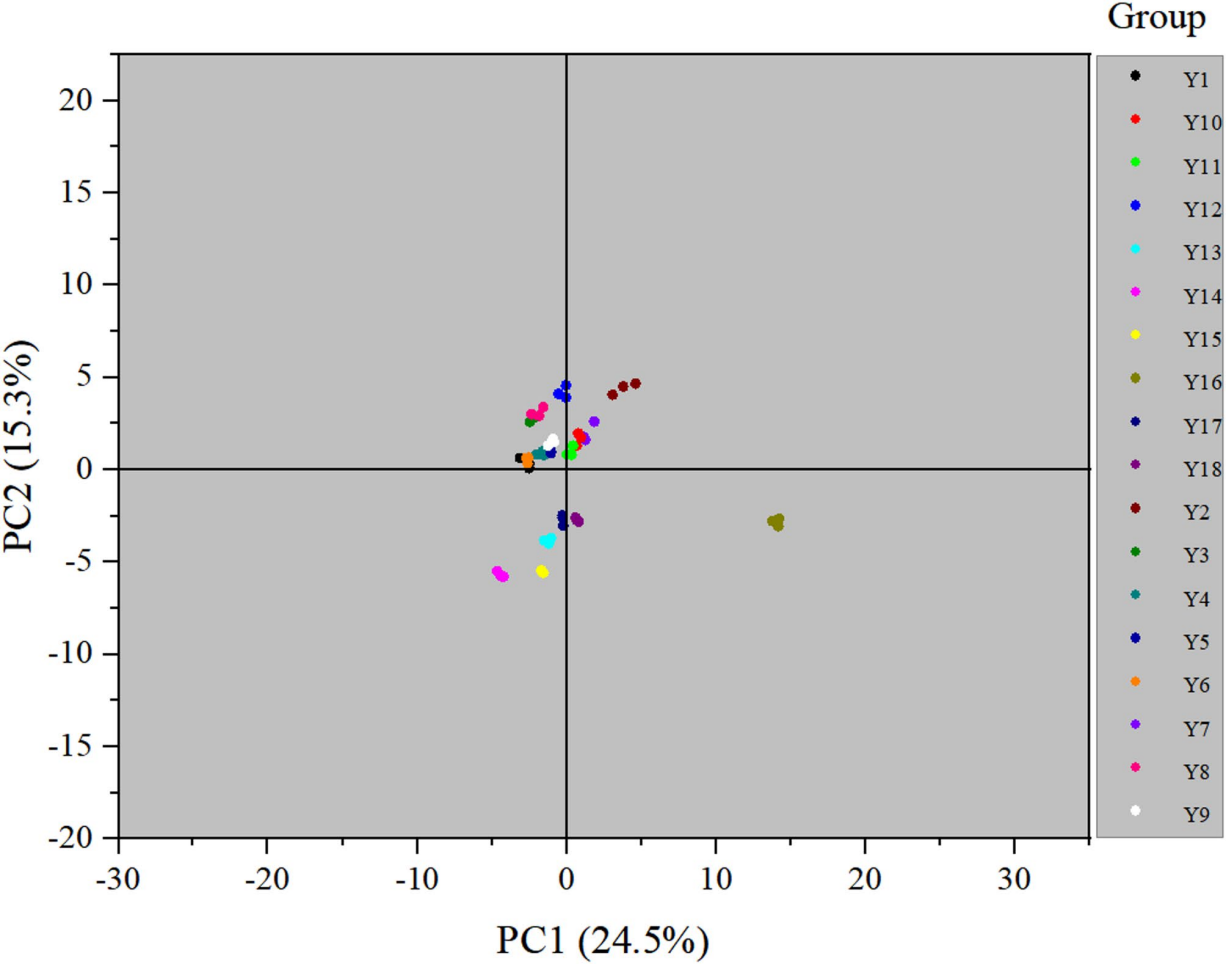
PCA of nutritional compositions and organic acids of the 18 pepper varieties. Y1-Y18 represent the numbers of 18 pepper varieties.

PCA was carried out on nutritional compositions and organic acids of the 18 pepper varieties examined to determine the correlations in differences (Fig. 3). The variance contribution rate of the first two principal components reached 39.8% (24.5% and 15.3% for PC1 and PC2, respectively). As a result, the varieties were split into three categories: Y16 formed one category; Y1-12 in the second quadrant were another; and all remaining varieties formed the last category.

### Heat map analysis of correlation each metabolite in 18 pepper varieties

**Fig. 4:**
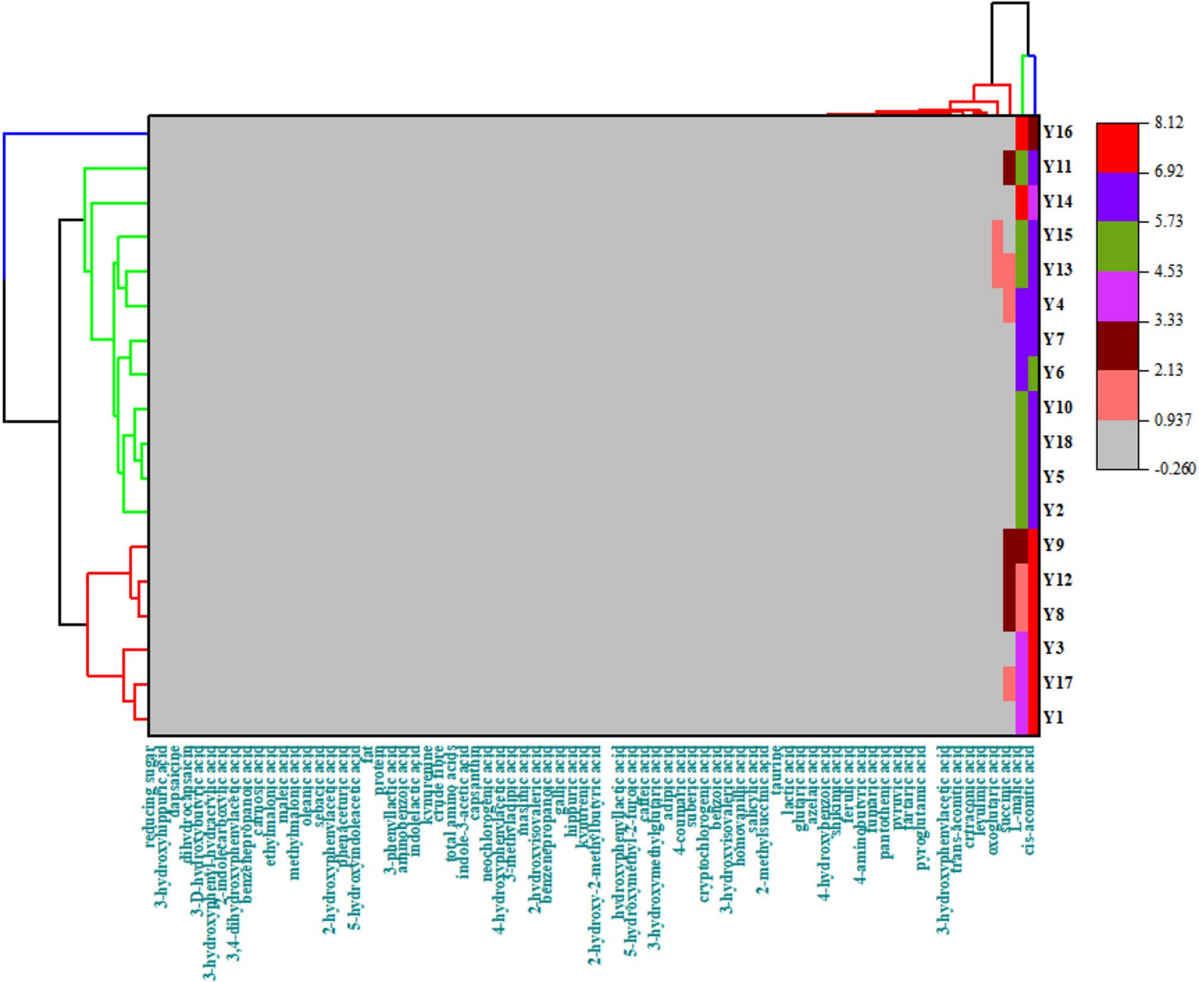
HCA between nutritional compositions and organic acids of the 18 pepper varieties. In the hierarchical clustering diagram, the color gradient from gray to red indicates a decreasing order of the measurement index values.

HCA was conducted on the nutritional compositions and measured the metabolite levels of all pepper varieties (Fig. 4), producing three general categories: Y16 was in one category, notably consistent with the PCA results; Y2, Y5, Y18, Y10, Y6, Y7, Y4, Y13, Y15,Y14 and Y11 in another, and the remaining varieties in the last category.

HCA was conducted on the nutritional compositions and measured the metabolite levels of all pepper varieties (Fig. 4), producing three general categories: Y16 was in one category, notably consistent with the PCA results; Y2, Y5, Y18, Y10, Y6, Y7, Y4, Y13, Y15,Y14 and Y11 in another, and the remaining varieties in the last category.

## Discussion

Plant proteins are crucial for the nutritional quality, affecting consumer choices. They provide essential amino acids for muscle growth and metabolism, while also impacting the texture and flavor^31^. The protein content of the 18 pepper varieties ranged from 9.96% to 14.4% (Fig. 1a)^32^. reported that the total soluble sugar content varied from 11.73% to 20.96%. In comparison, the change range of protein in the pepper varieties explored here was greater, likely due to the influence of variety, fruit maturity, cultivation area^33,34^. In the context of chili pepper breeding, high-protein varieties(Y6 and Y8) were developed as breeding materials to meet the demand for nutritious food.

Amino acids are essential for human health and required for the growth, development, regeneration and reconstruction of the body and are responsible for the production of antibodies, blood cells, hormones and enzymes^35^. The total amino acid content of the 18 pepper varieties varied from 9.53% to 34.48% (Fig. 1b). Pepper varieties lead to the larger range, probably. Different pepper materials could be selected for amino acids in pepper breeding.

Fat and fiber are important nutrients. Fat serves as a source of energy and facilitated the release of functional components^36^. Fiber can promote the excretion of bile acids and reduce the synthesis of cholesterol in the liver^37^. In our studies, the fat and fiber content of the pepper varieties were 9.23%−9.5%, 19.77%−32.64% respectively. Similar results for red pepper were reported by earlier studies^38–41^. However, a study conducted in 2021 by Collera et al.^42^ showed the crude fiber content in the Addis Ababa chili variety was 44% significantly higher than our findings. The variations in proximate compositions may be attributed to the factors such as growing conditions, variety, season, differences in production and processing methods, and storage practices or analytical methods used.

Capsaicin and dihydrocapsaicin are unique substance in pepper fruit, and was characterized by a strong spicy taste and positive health stimulation^43,44^. They exhibit various pharmacological effects, including antioxidant, anti-inflammatory, antibacterial, and energy metabolism-enhancing properties. These compounds can serve as natural antioxidants and inhibitors of fat accumulation, thereby contributing to a reduction in the risks associated with cardiovascular diseases and cancer^18^. Among the 18 varieties, the content of capsaicin and dihydrocapsaicin changed greatly. The difference in capsaicin content between the variety with the highest content (Y16: 6.86 mg g^− 1^) and that with the lowest (Y4: 0.15 mg g^− 1^) exceeded 45 times. High capsaicin varieties, such as Y16, were particularly well-suited for the development of functional foods that may aid in weight management and serve as adjunct treatments for neurodegenerative diseases. Accordingly, it was observed that varieties with higher capsaicin content also exhibited correspondingly higher dihydrocapsaicin levels, The finding consistent with the result reported by Li J et al.^18^.

Capsanthin, a natural red-orange pigment found in red paprika, has been proven to have various health benefits^45^. The capsanthin content varied from 11.63 mg g^− 1^ to 47.73 mg g^− 1^ in our study. Unlike our studies^32^, reported that the capsanthin content was 4.6 mg g^− 1^ to 7.81 mg g^− 1^. The pepper varieties maybe make a difference. The reducing sugar content varied from 0.73% to 4.17% in our study, whereas^32^ reported that the it was 6.61%−18.71%. The discrepancies may be influenced by the factors such as variety, cultivation region, storage time and conditions.

Organic acids are an important component in foods and contribute to the palatability of foods^31^. The absence of 11 organic acids among the 18 pepper varieties indicated that these peppers was not involved in the metabolism of these specific organic acids. The results implied that the composition of organic acids might vary depending on the cultivar. Some studies^18,46–48^ have concentrated on a limited selection of organic acids(malic acid, ascorbic acid, citric acid, tartaric, oxalic acid, fumaric acid, Succinic acid) and few studies were conducted on other organic acids. To the best of our knowledge, this study represents the first comprehensive analysis of the organic acid profiles of various pepper varieties, providing a comparative overview of these profile. In this paper, 64 organic acids were analyzed, which enriched the research of organic acids in peppers.

Among the detected organic acid components, cis-aconitic acid is the most abundant across the 18 pepper varieties, exhibiting a wide range of variation. The difference between the varieties with the highest and the lowest content is as much as 6.39 times. Following closely behind cis-aconitic acid in content is L-malic acid, showing a noteworthy similar with the results of reference^18^, and the highest value is 3.24 times the minimum value. According to Luning^49^, malic acid is dominant in green pepper, whereas citric acid is major in the mature red stage. The pepper materials we studied belong to dry peppers, L-malic acid is dominant organic acid component across varieties.

PCA is a multivariate statistical method that can be used to investigate the correlations between multiple variables^18,50^. used PCA to classify and distinguish amino acid and polyphenol components in 69 cabbage varieties. Here, PCA was conducted for the organic acids of the 18 pepper varieties, revealing that a total variance contribution rate of 39.8% for the two principal components. This indicated a limited capacity to reflect the information of the original variables. The varieties classified grouped together indicated greater similarity in their nutritional compositions and organic acid components, while those not classified in the same group showed differences in characteristics of detection indicators. The 18 varieties were broadly classified into three categories: Y16 formed one category; Y1-12 in the second quadrant were another; and all remaining varieties formed the last category. The result that Y16 formed one category was consistent with the result of the HCA, where the clustering of varieties can largely be explained by the relatively similar nutritional compositions and organic acids of these samples.

## Conclusions

Through the analysis of nutritional traits and organic acids in 18 pepper varieties, this study revealed notable differences in these characteristics. In terms of total amino acid, crude fiber, capsaicin, and dihydrocapsaicin, variety Y16 exhibited the highest content. For fat, capsanthin, and reducing sugars, varieties Y13, Y9, and Y14 reached their maximum values, respectively. A total of 53 organic acids were identified, with cis-aconitic acid, L-malic acid, and succinic acid constituting 77.5% to 86.7% of the total organic acids detected. Significant inter-variety differences in organic acid composition were observed, with the content of 2-hydroxyisovaleric acid differing by a factor of 12.36, indicating a rich metabolic diversity of organic acids in peppers. However, this study primarily focused on the analysis of nutritional components and organic acids, employing principal component analysis (PCA) and hierarchical cluster analysis (HCA) for variety classification. It did not investigate the molecular regulatory mechanisms underlying the differences in nutritional traits among varieties. In subsequent studies, we will broaden the scope of test materials to encompass additional pepper species and wild germplasm, and will integrate transcriptome and metabolome analyses, to elucidate the molecular basis of nutritional differences, thereby providing more comprehensive theoretical support for pepper quality breeding.

## Supplementary Information

Below is the link to the electronic supplementary material.

Supplementary Material 1

Supplementary Material 2

Supplementary Material 3

## Data Availability

The datasets used and/or analyzed during the current study are available from the corresponding author on reasonable request.
