# Supplementary material for: A comparative study on the nutrient and organic acid profiles of selected pepper genotypes
Source: Sci Rep. 2026 Jan 28;16:6435. doi: 10.1038/s41598-026-37078-w (PMC12909785; doi:10.1038/s41598-026-37078-w)
Supplement: Supplementary file 2 — Supplementary Material 2 [file 41598_2026_37078_MOESM2_ESM.docx]

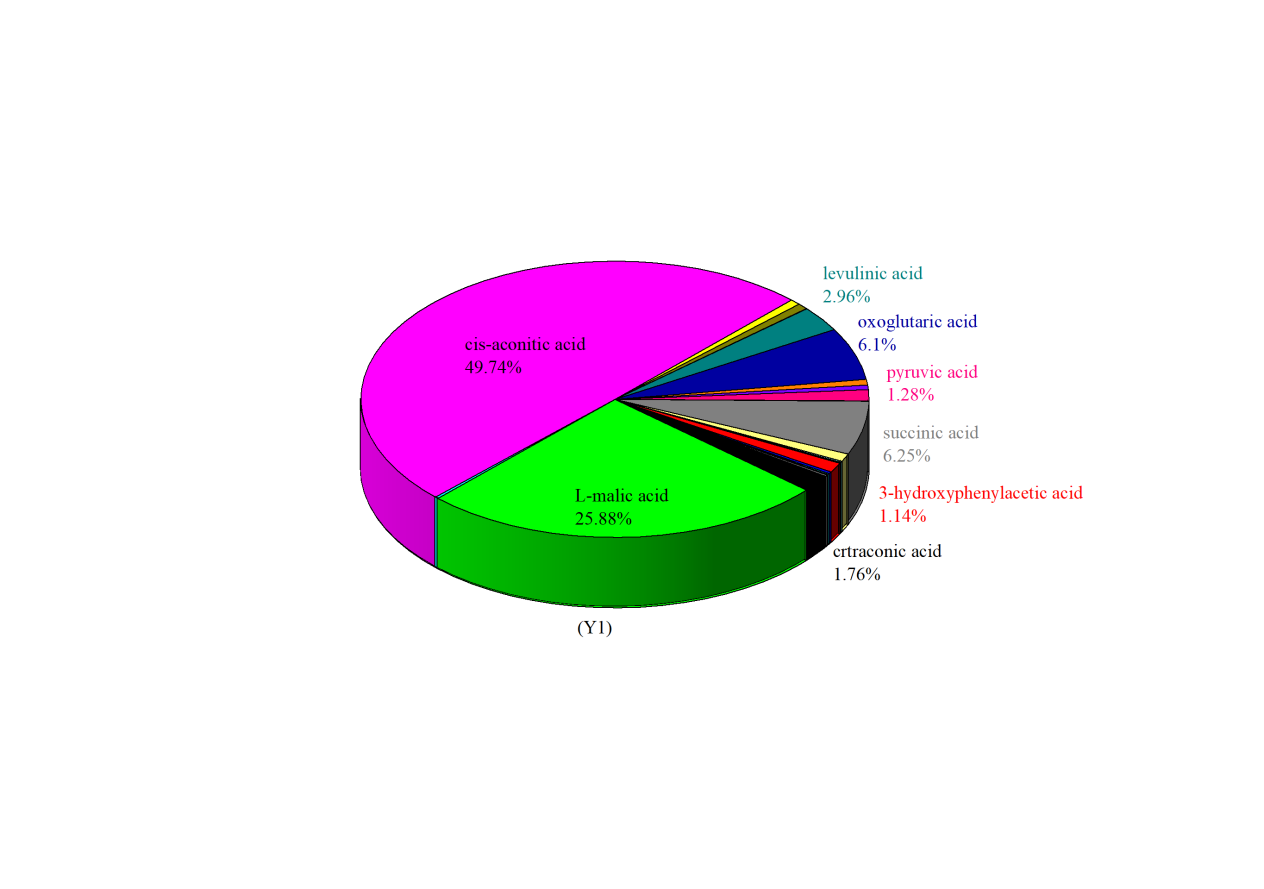


Fig. S1. Contents and Proportions of Major Organic Acids in Sample Y1.


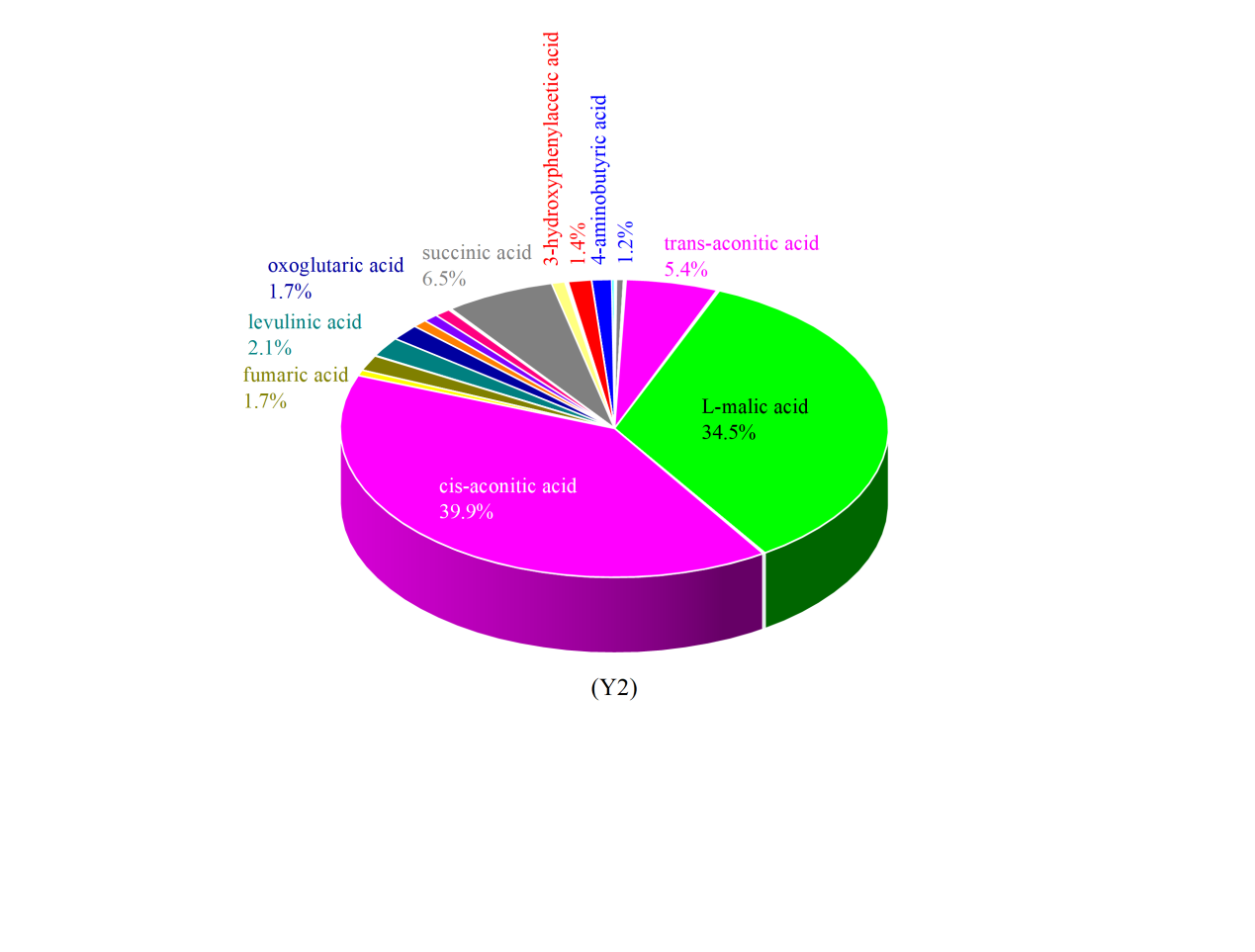


Fig. S2. Contents and Proportions of Major Organic Acids in Sample Y2.


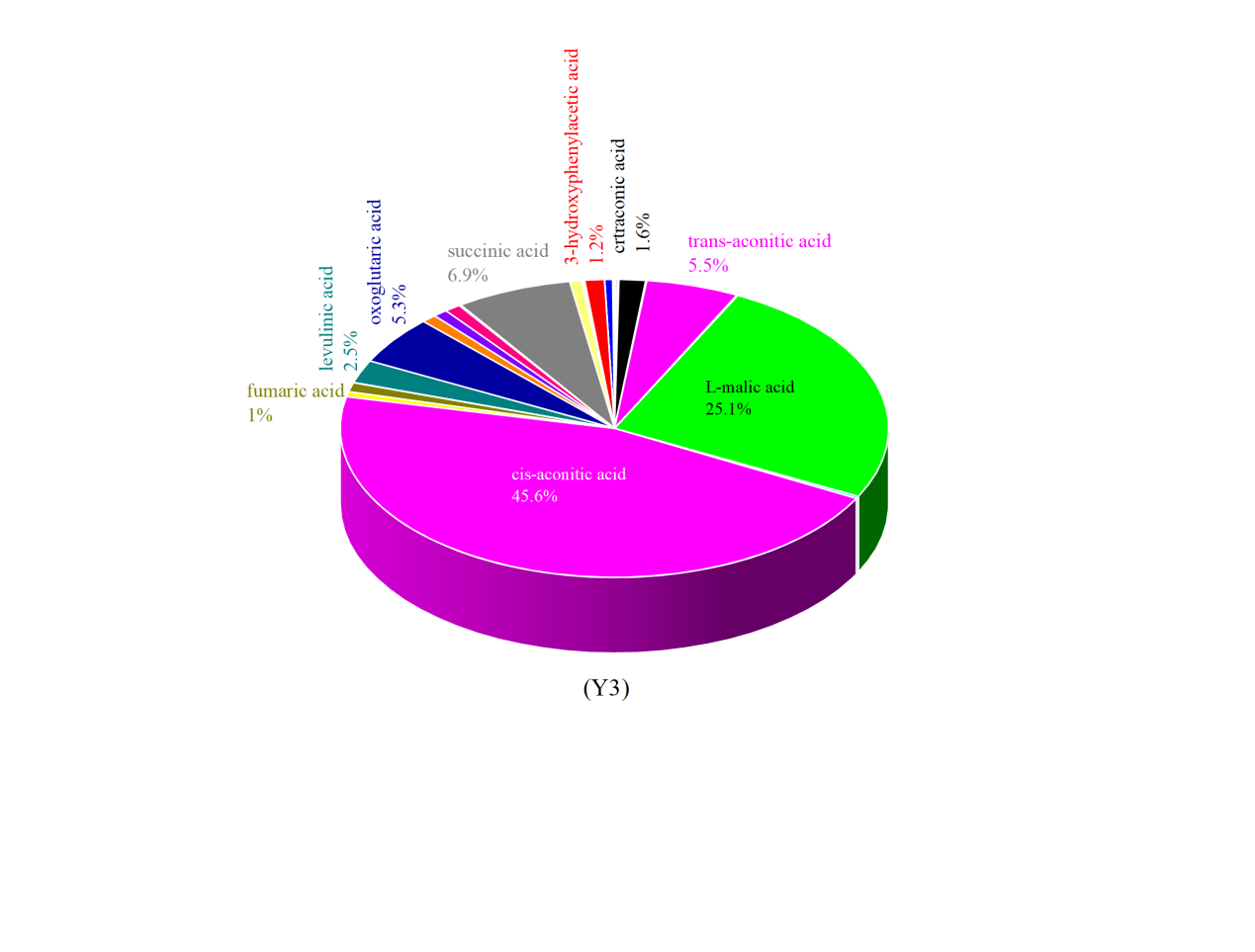


Fig. S3. Contents and Proportions of Major Organic Acids in Sample Y3.


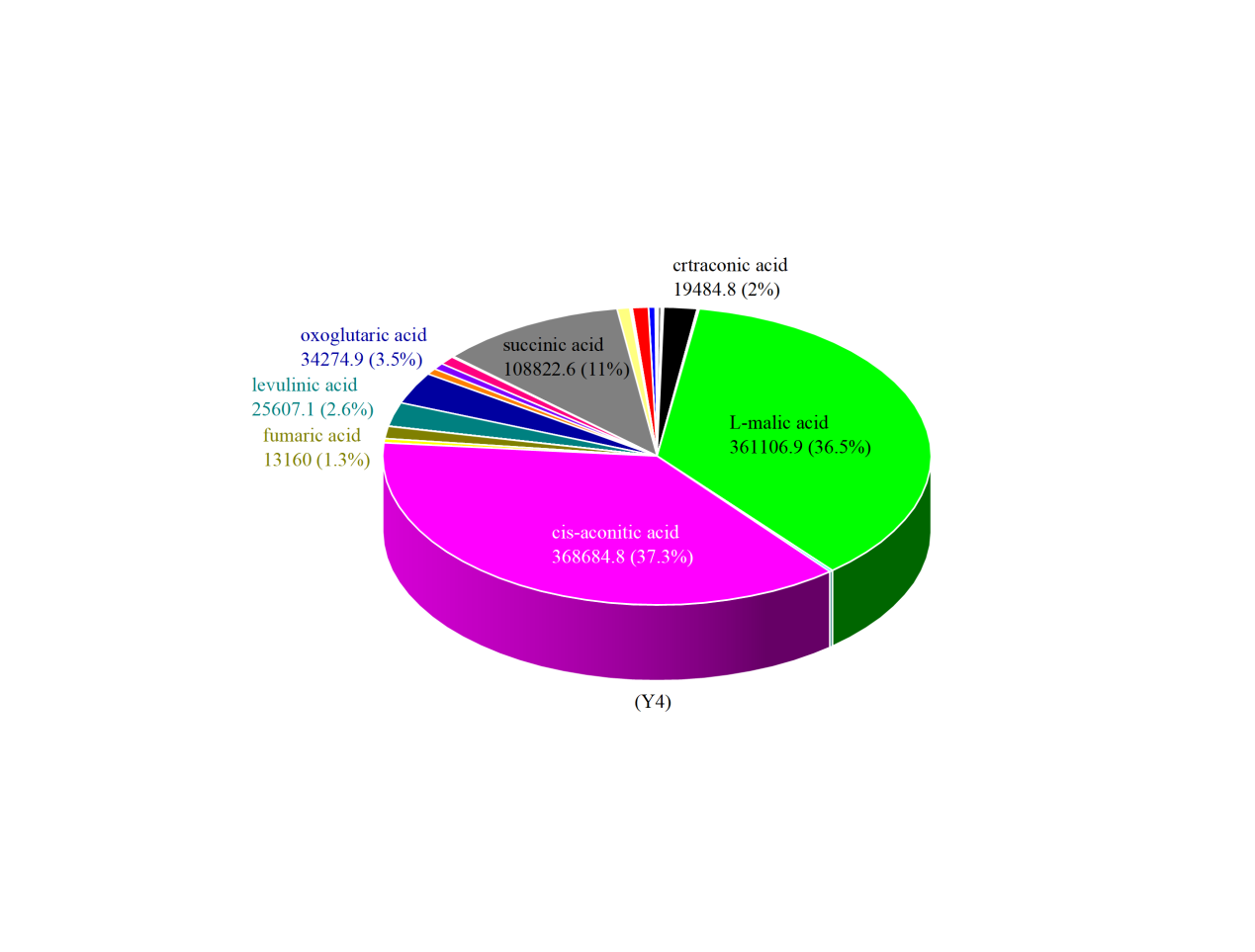


Fig. S4. Contents and Proportions of Major Organic Acids in Sample Y4.


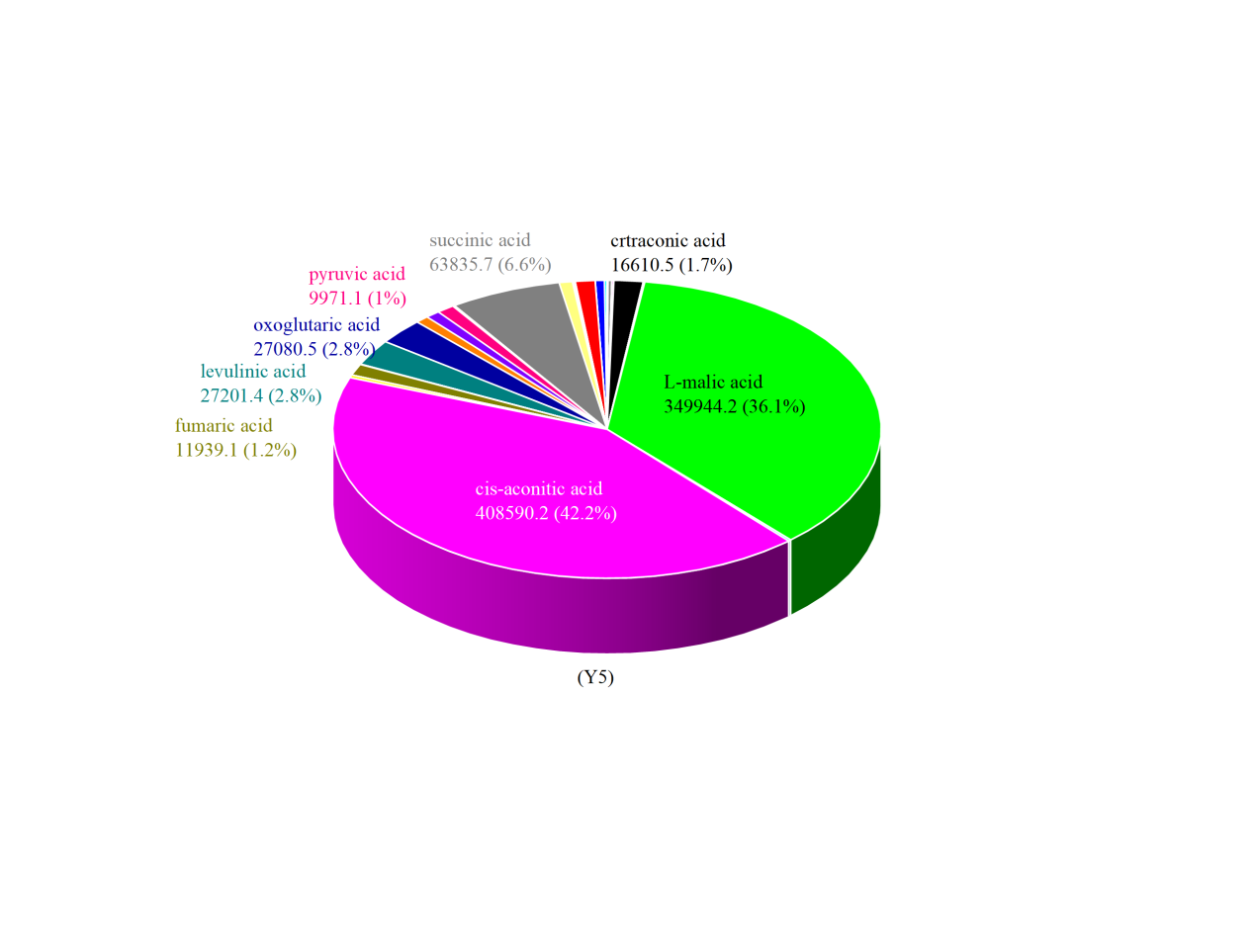


Fig. S5. Contents and Proportions of Major Organic Acids in Sample Y5.


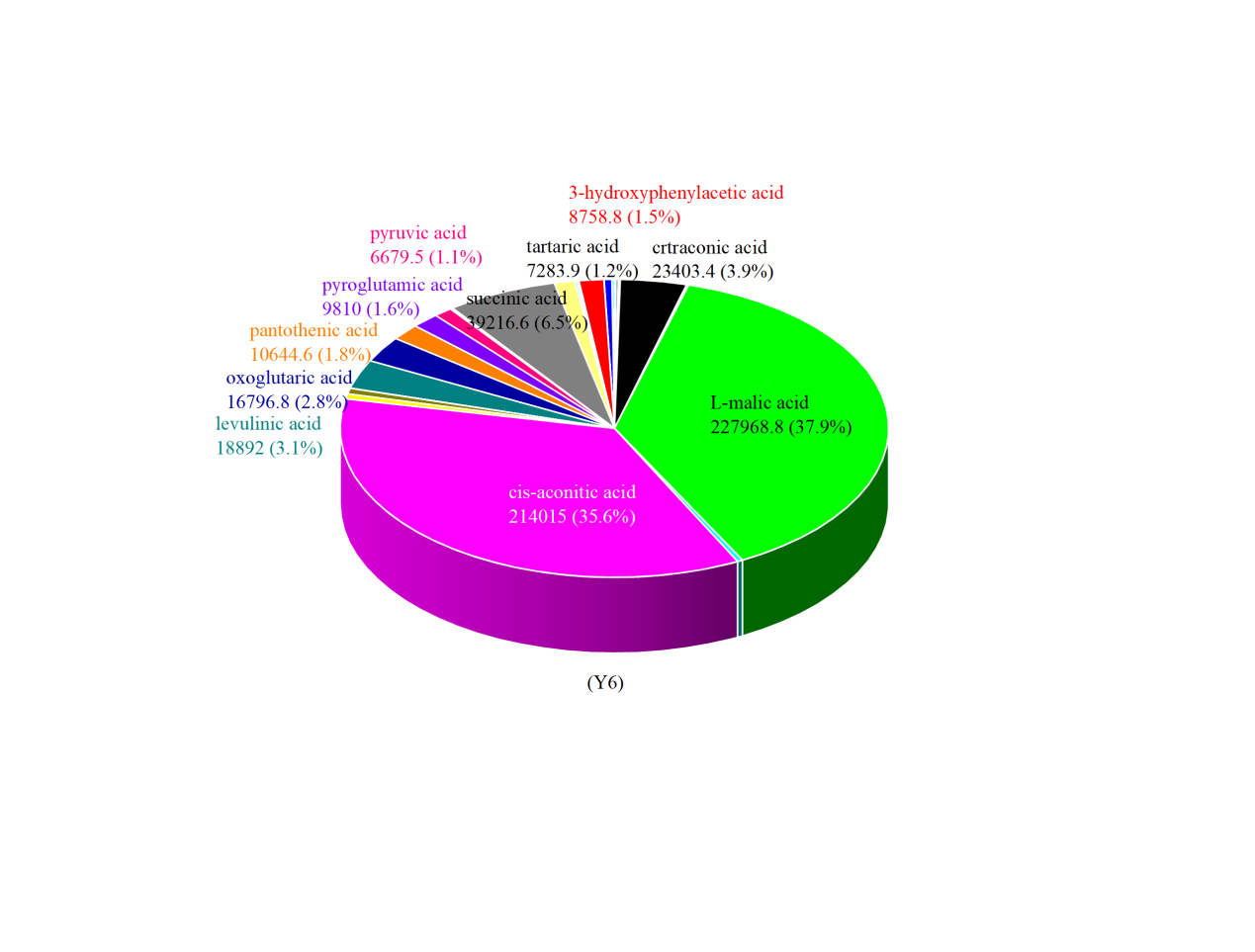


Fig. S6. Contents and Proportions of Major Organic Acids in Sample Y6.


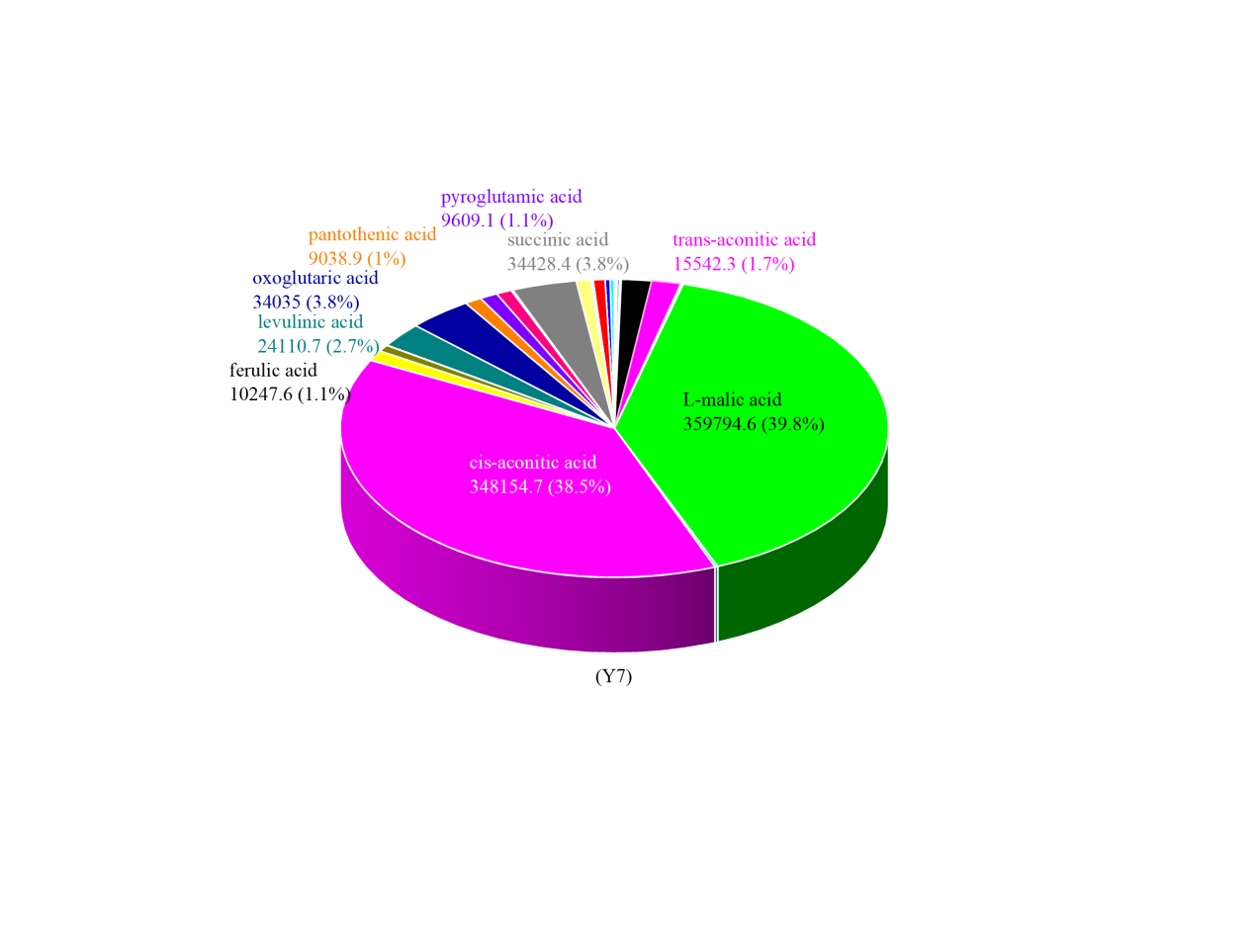


Fig. S7. Contents and Proportions of Major Organic Acids in Sample Y7.


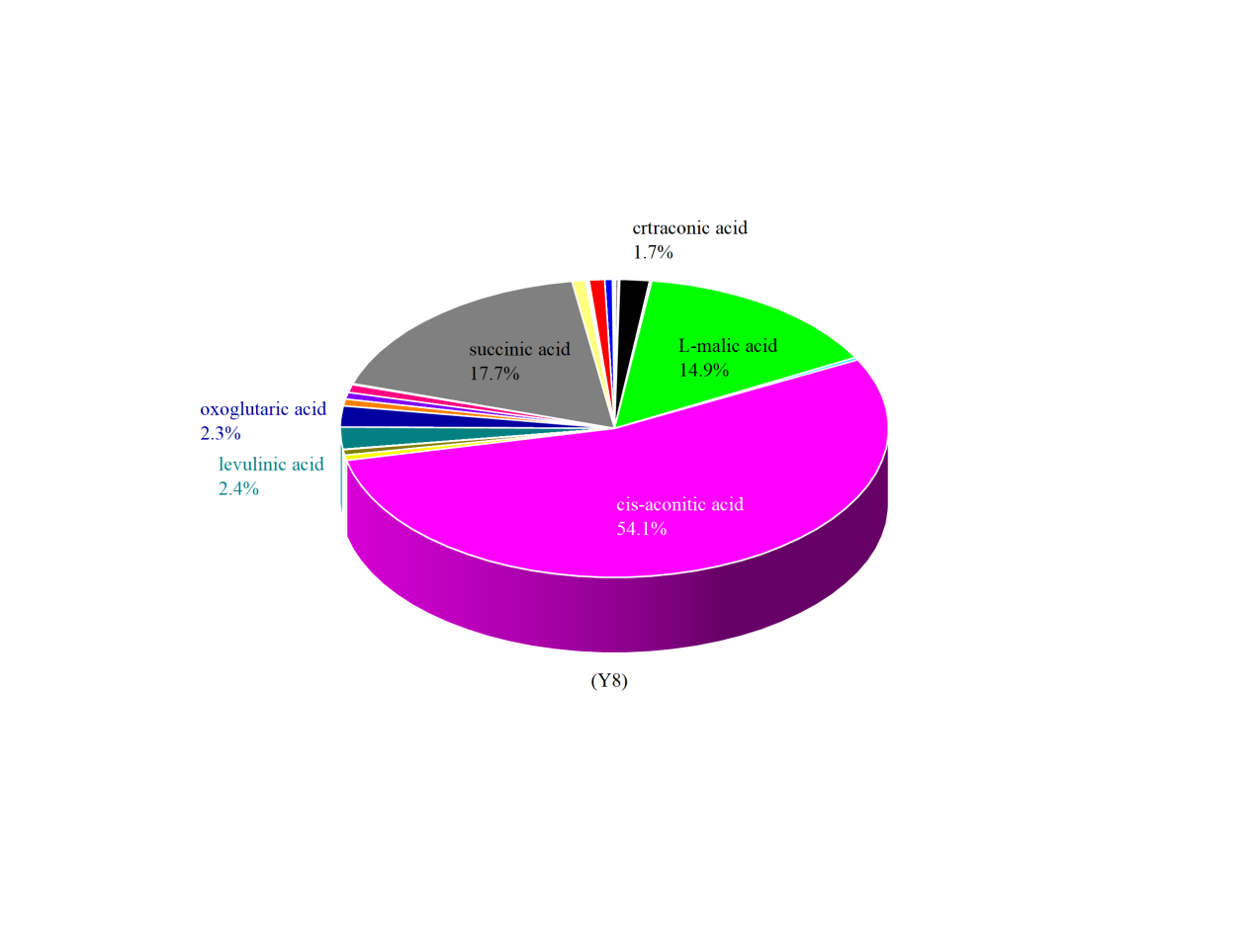


Fig. S8. Contents and Proportions of Major Organic Acids in Sample Y8.


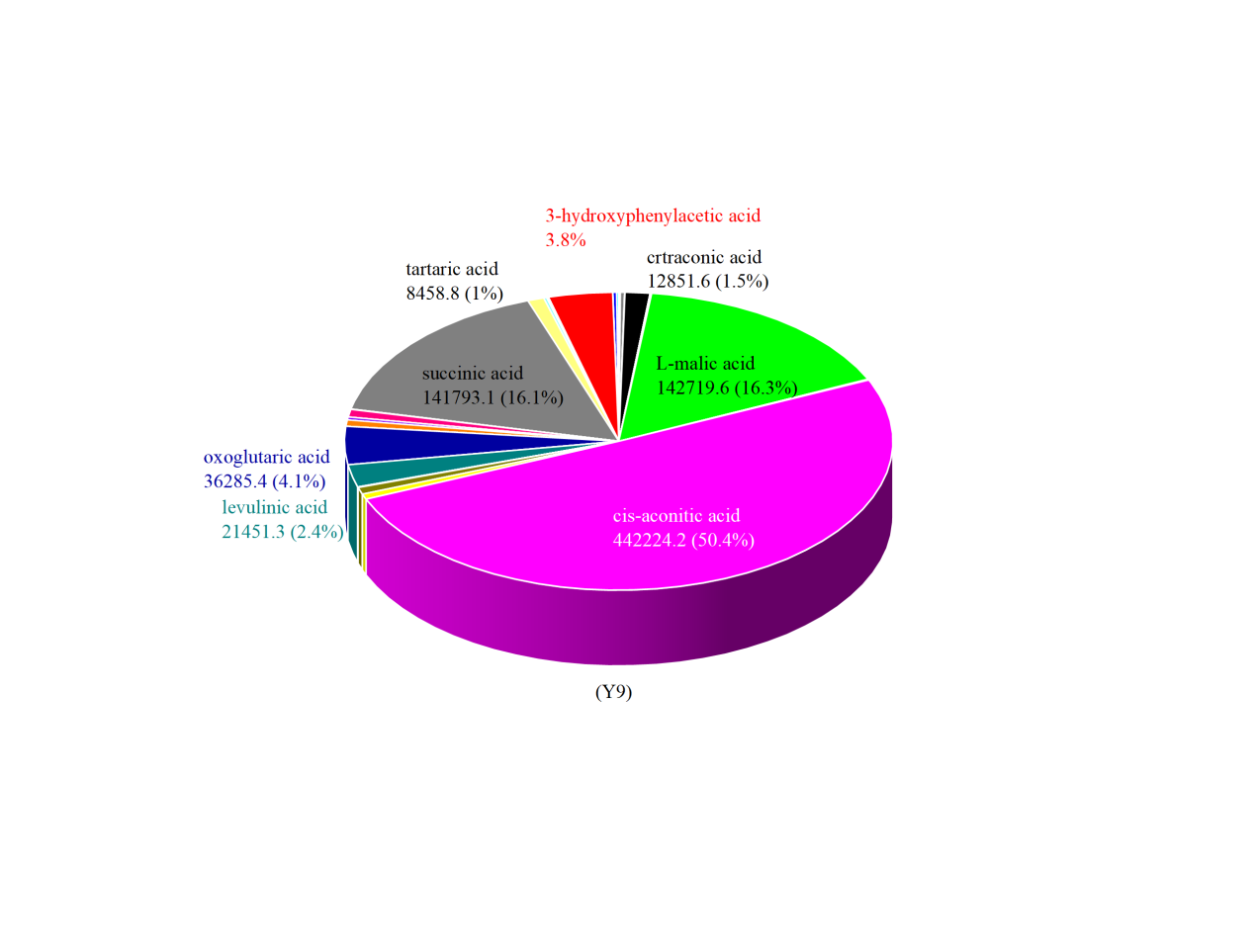


Fig. S9. Contents and Proportions of Major Organic Acids in Sample Y9.


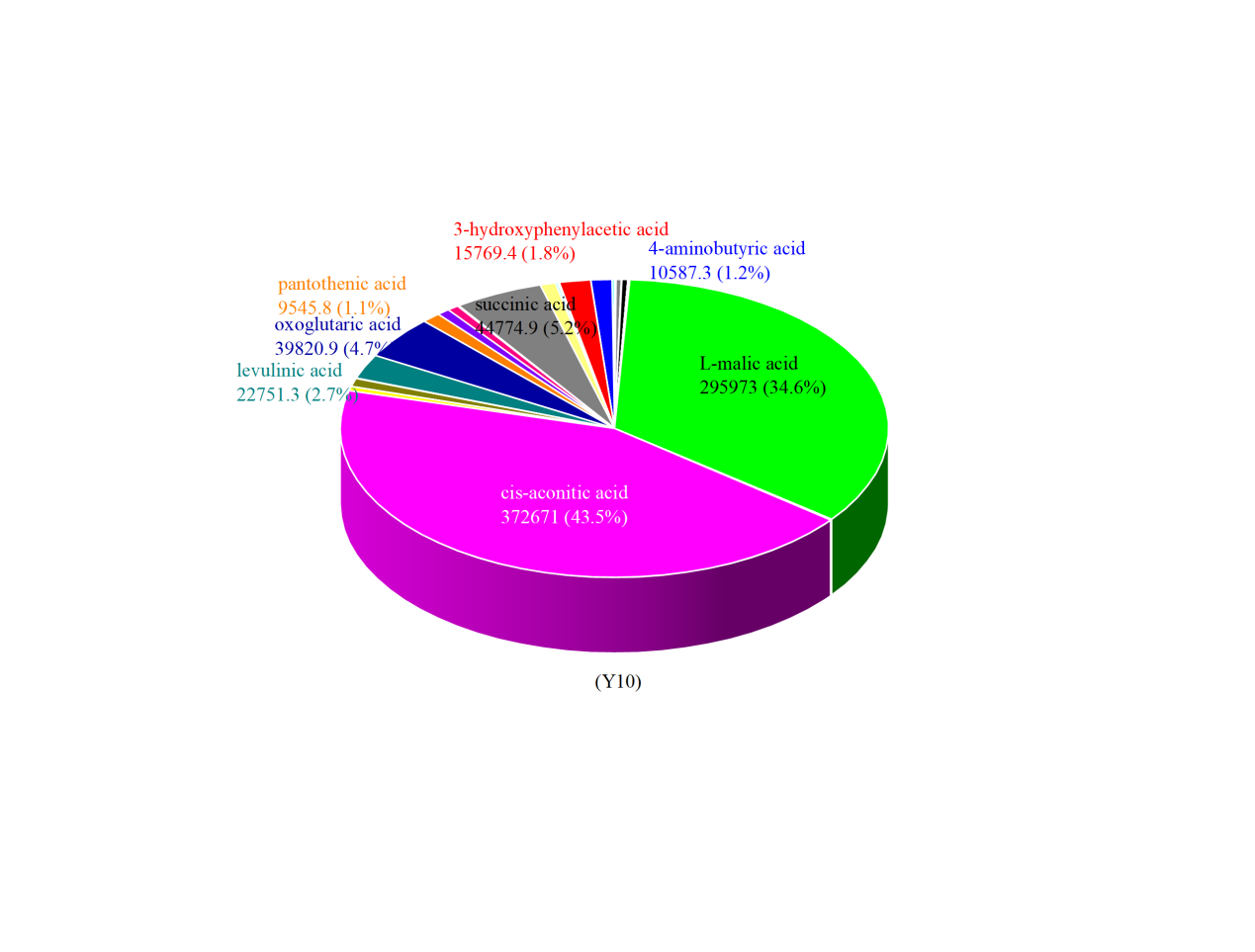


Fig. S10. Major organic acids content of Y10 sample.


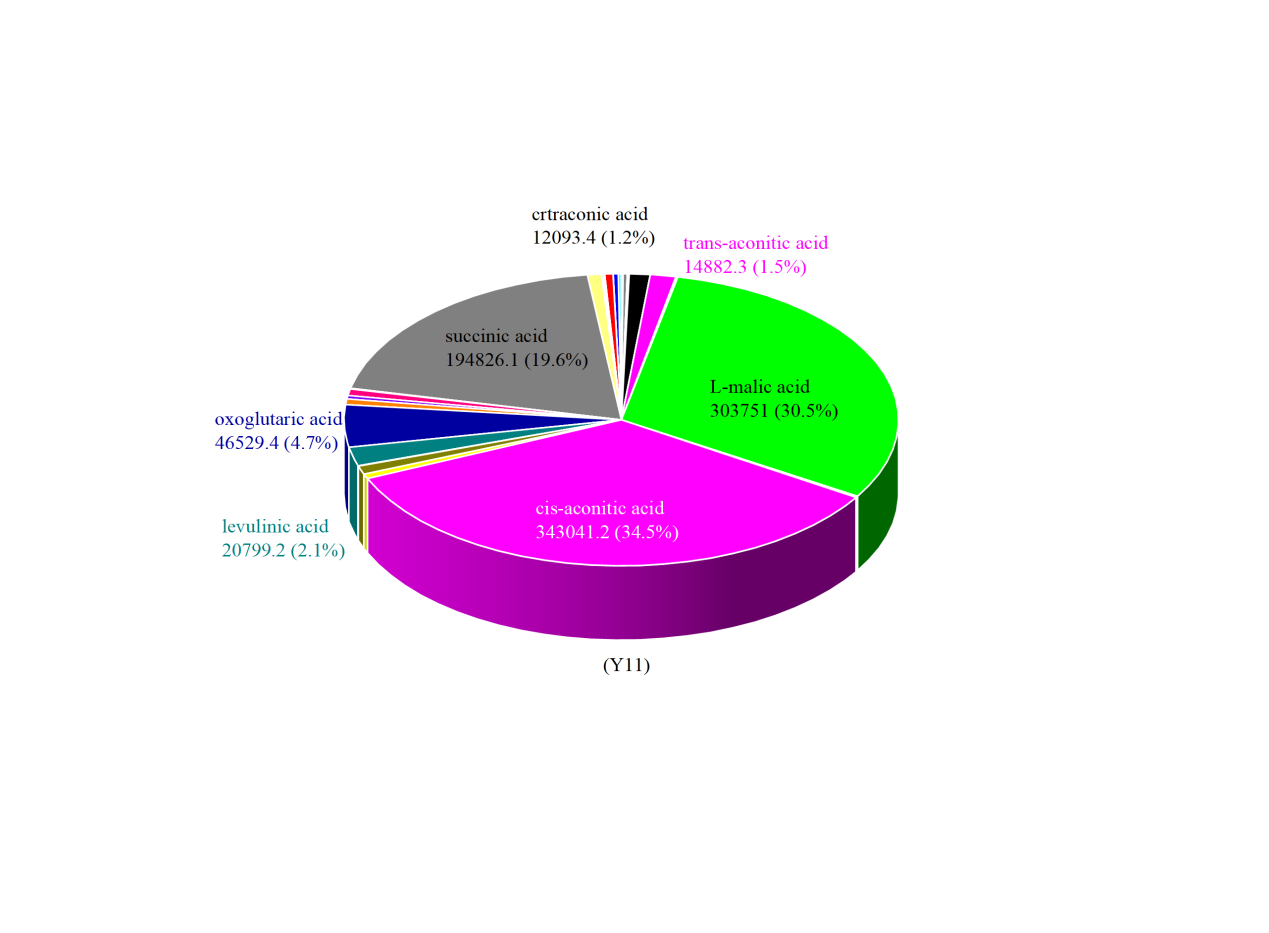


Fig. S11. Contents and Proportions of Major Organic Acids in Sample Y11.


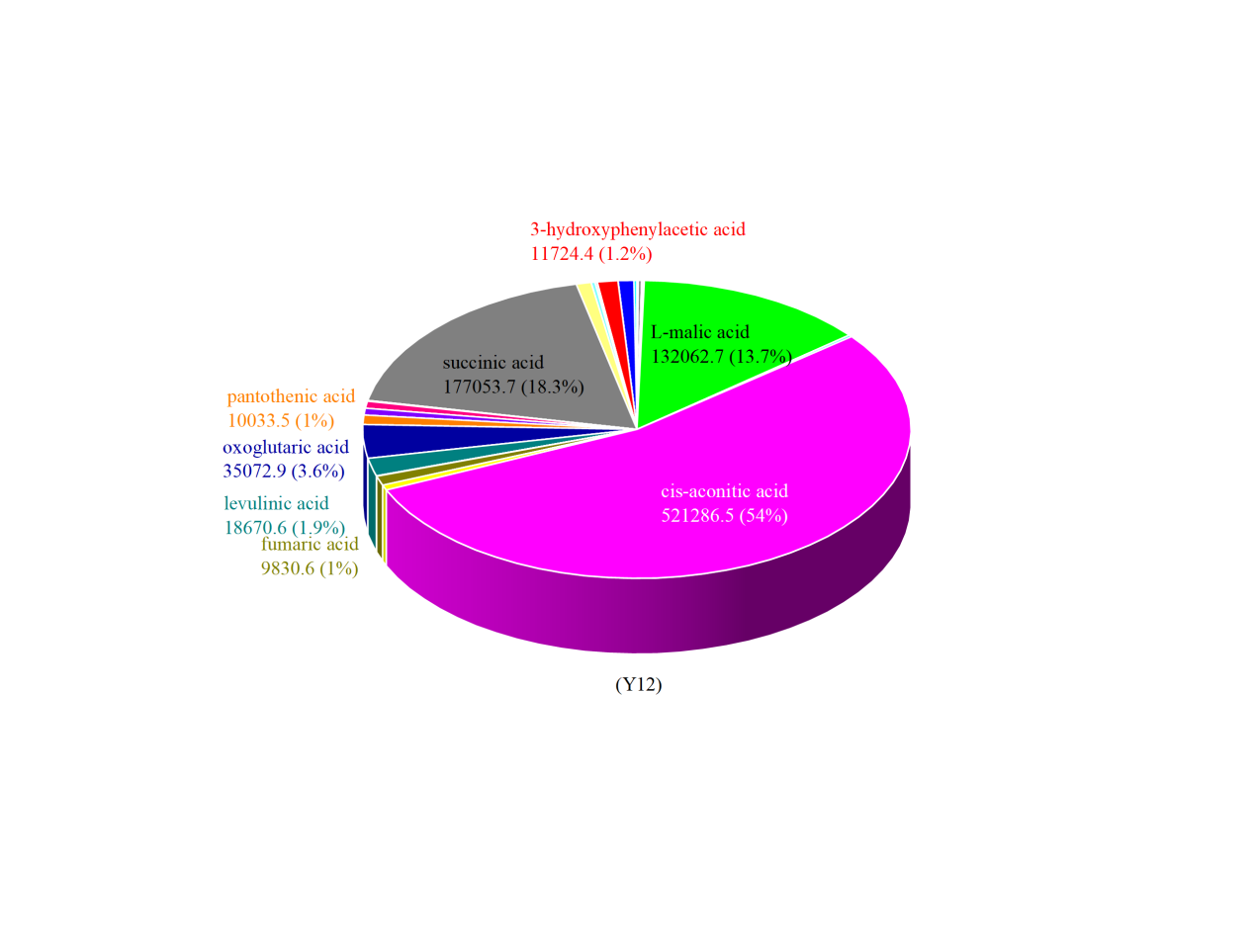


Fig. S12.Contents and Proportions of Major Organic Acids in Sample Y12.


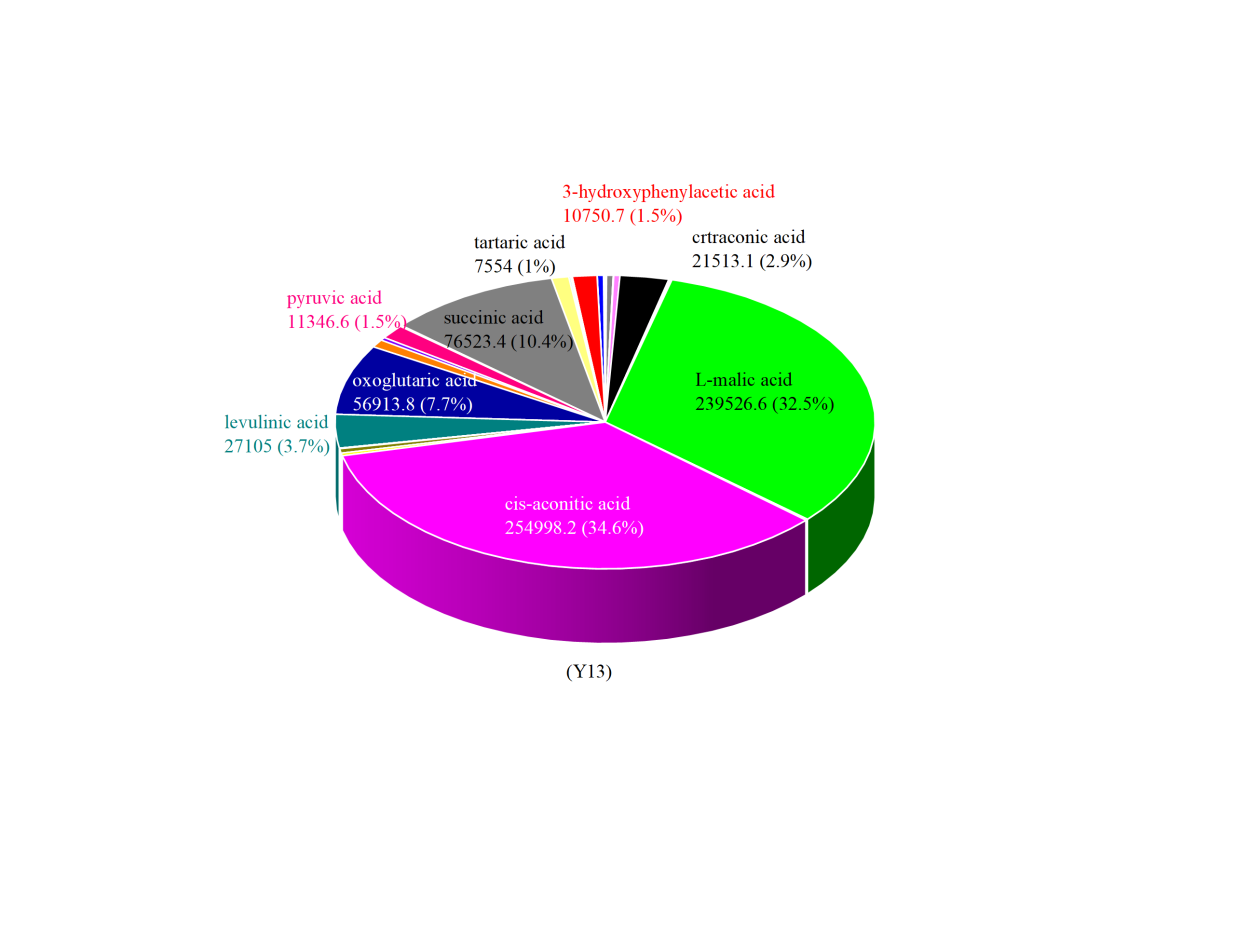


Fig. S13. Contents and Proportions of Major Organic Acids in Sample Y13.


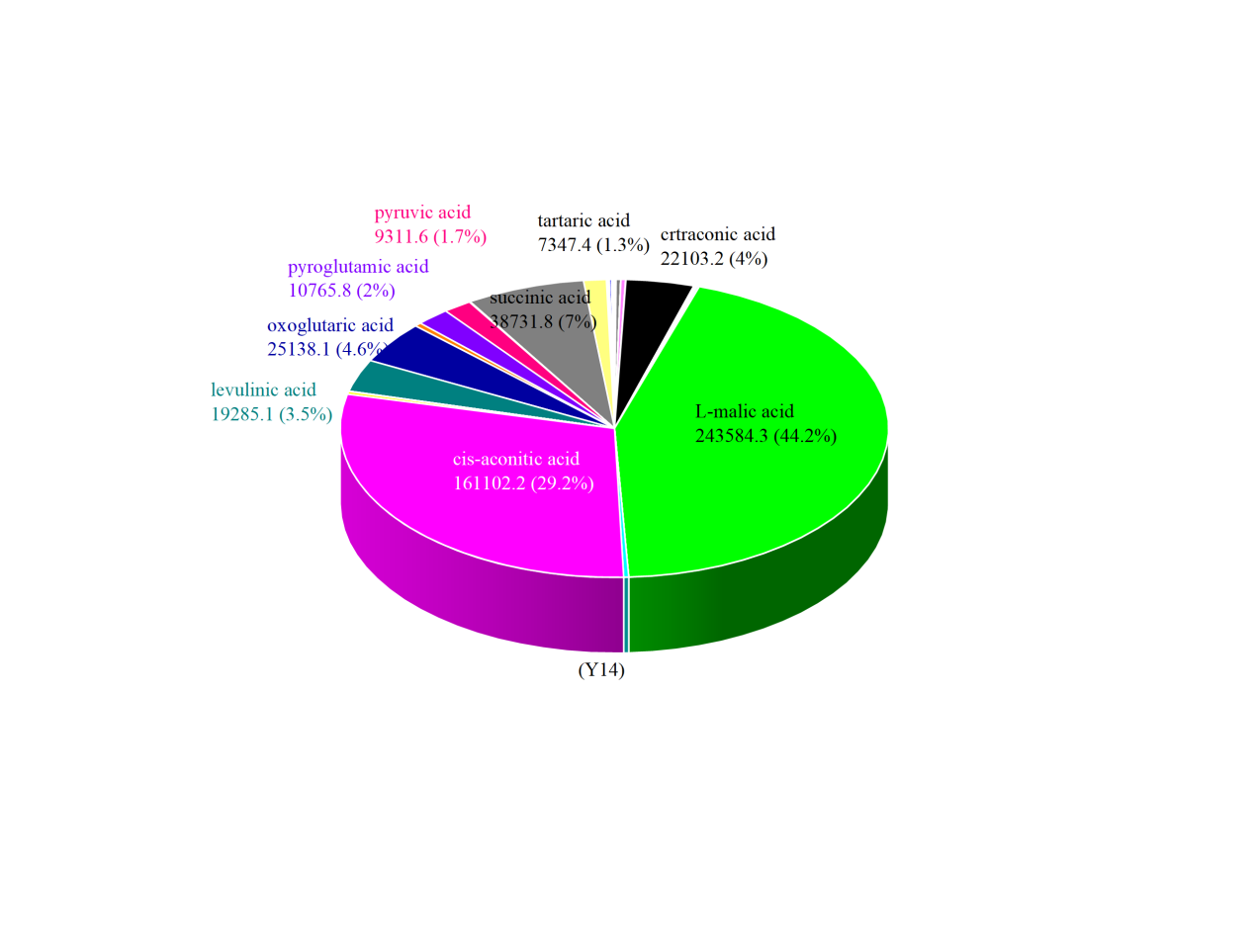
Fig. S14. Contents and Proportions of Major Organic Acids in Sample Y14.


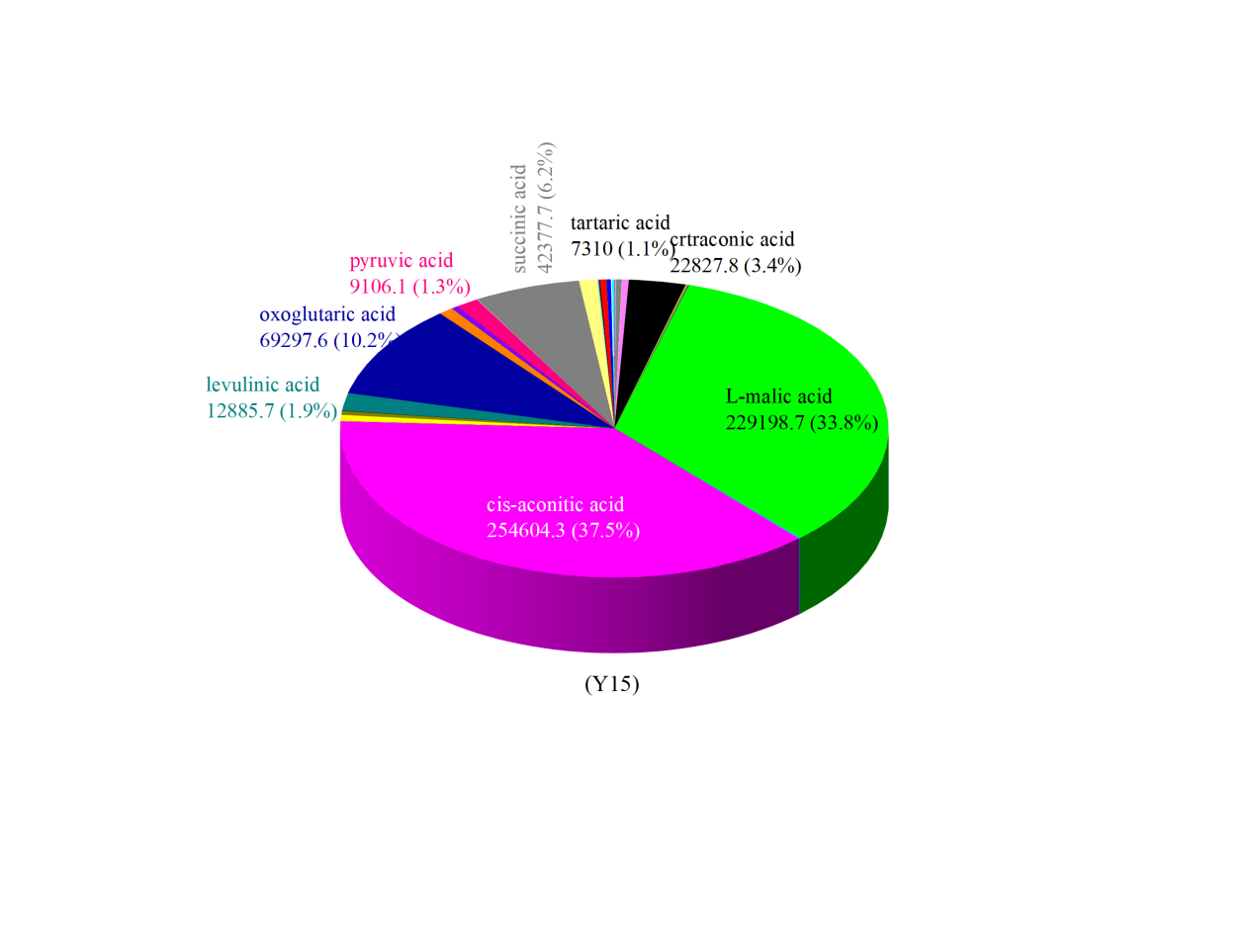


Fig. S15. Contents and Proportions of Major Organic Acids in Sample Y15.


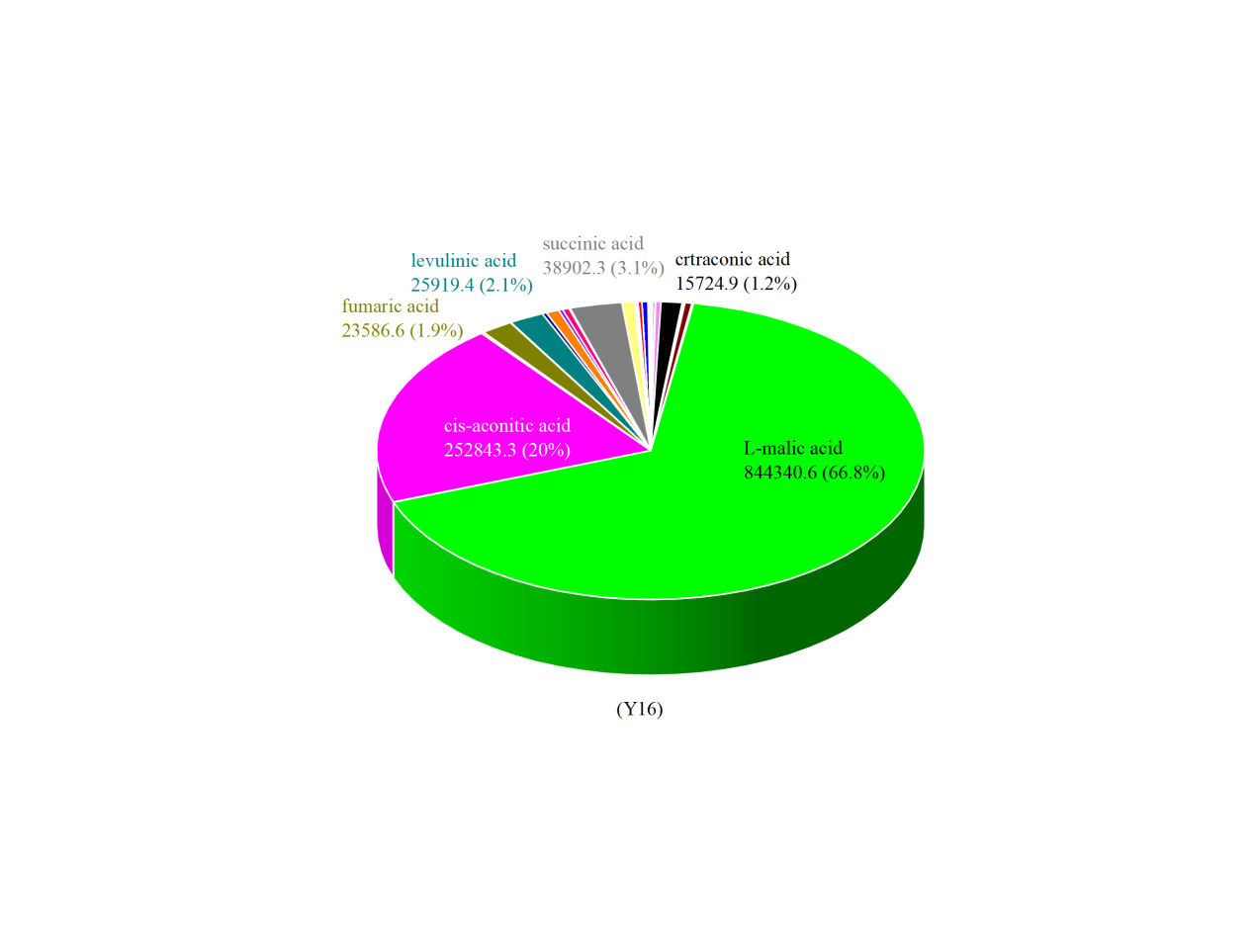


Fig. S16.Contents and Proportions of Major Organic Acids in Sample Y16.


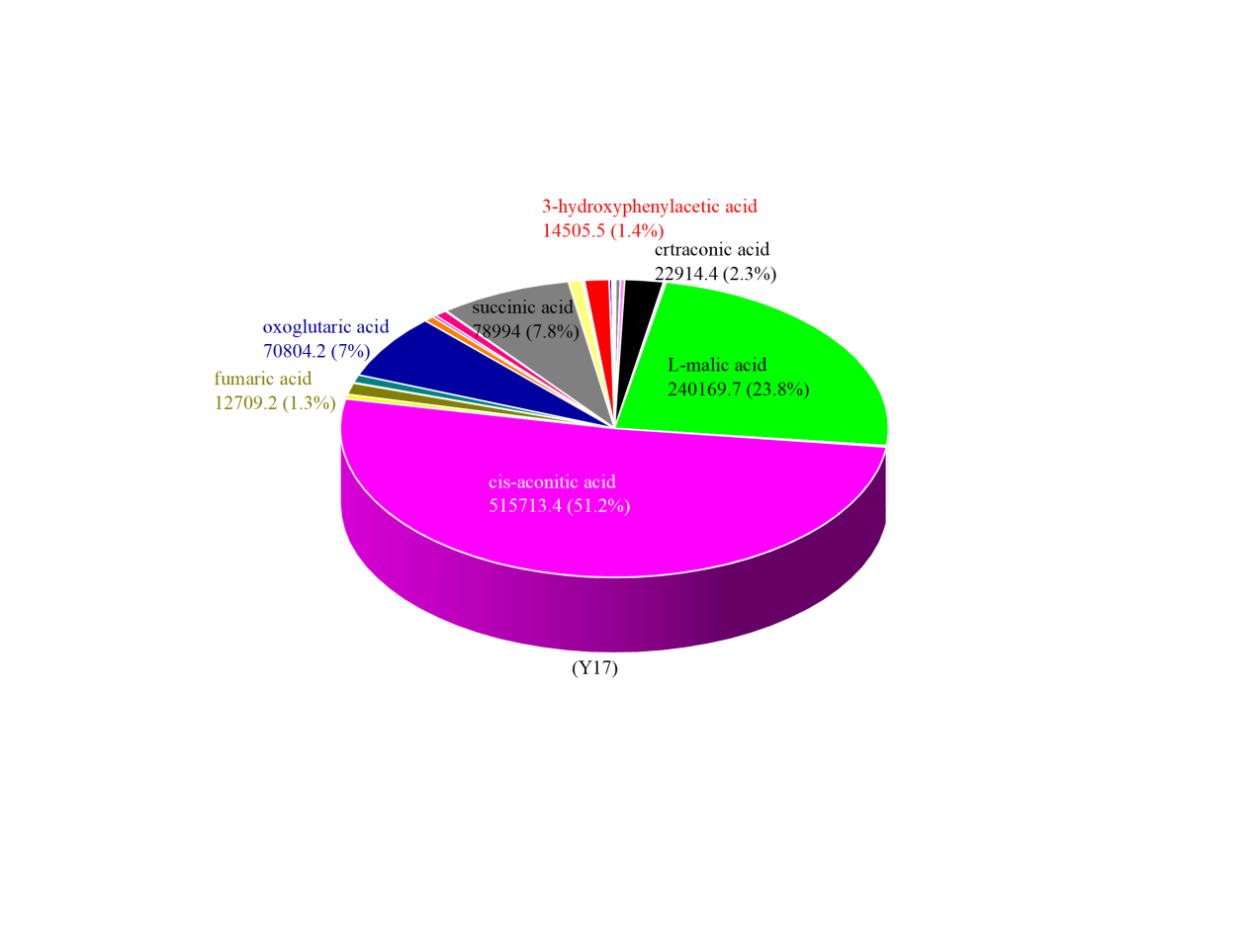


Fig. S17. Contents and Proportions of Major Organic Acids in Sample Y17.


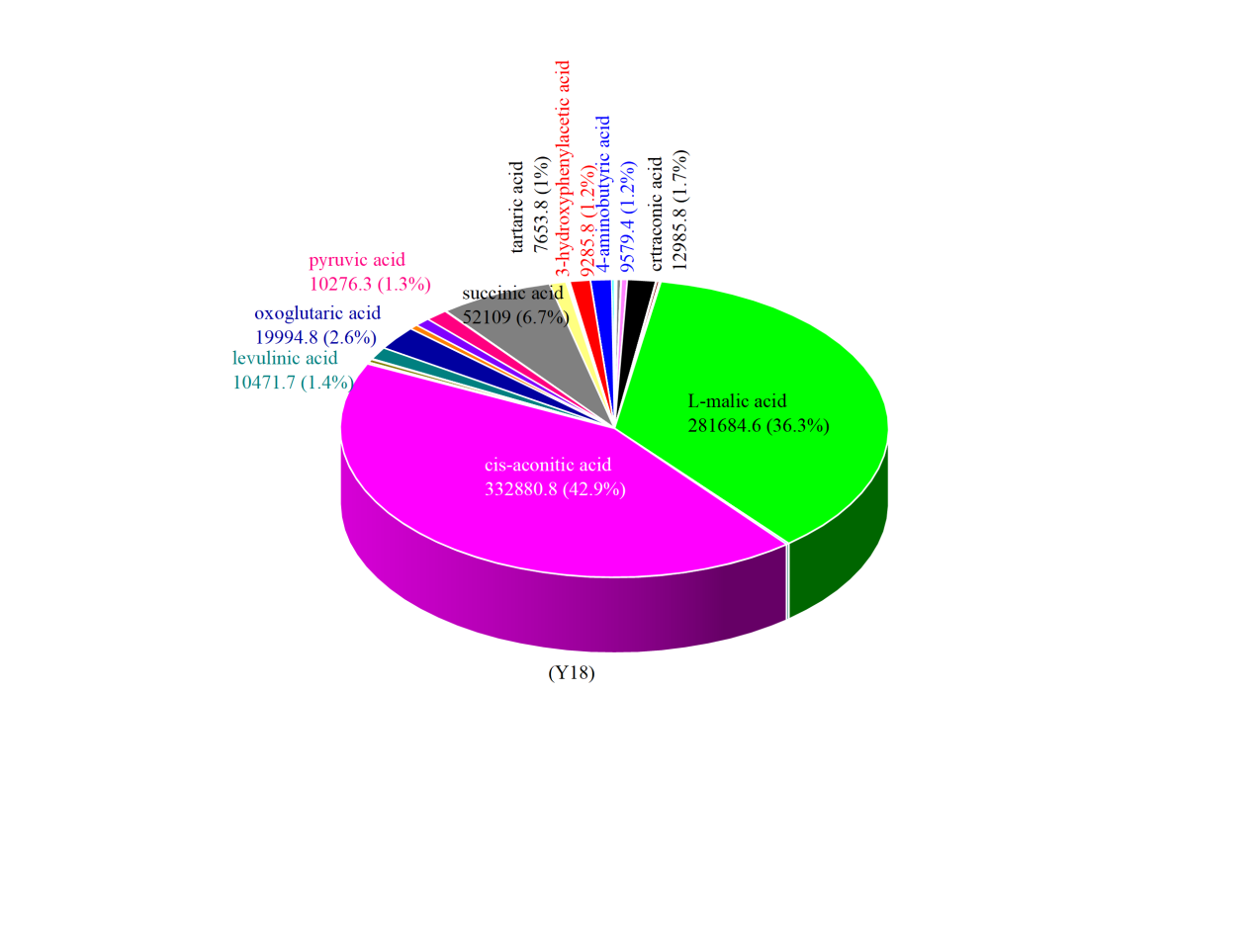


Fig. S18. Contents and Proportions of Major Organic Acids in Sample Y18.
